# Pyramiding elite alleles of the genetically linked *OsNRAMP5* and *OsHMA3* confers low Cd accumulation in rice grains without compromising stress tolerance

**DOI:** 10.1016/j.xplc.2025.101690

**Published:** 2025-12-30

**Authors:** Li Tang, Jiao Wang, Zhongying Ji, Xingrong Li, Xiaoshuang Liu, Qiming Lv, Pengcheng Wei, Xianlan Hu, Yaokui Li, Bigang Mao, Ye Shao, Yan Peng, Zhongwei Wei, Lianyang Bai, Caiyan Chen, Bingran Zhao

**Affiliations:** 1 State Key Laboratory of Hybrid Rice, Hunan Hybrid Rice Research Center, Hunan Academy of Agricultural Sciences, Changsha 410125, China; 2 Longping Agricultural College, Hunan University, Changsha 410125, China; 3 College of Agronomy, Anhui Agricultural University, Hefei 230036, China; 4 Institute of Subtropical Agriculture, Chinese Academy of Sciences, Changsha 410125, China; 5 Yuelushan Laboratory, Changsha 410128, China

**Keywords:** grain cadmium, manganese uptake, intracellular trafficking, NRAMP, VAMP-associated protein, allelic variation

## Abstract

Excessive cadmium (Cd) accumulation in rice grains poses a serious threat to food security. Cd uptake by roots and its subsequent transport to grains are mediated by mineral element transporters in rice. Consequently, efforts to reduce Cd accumulation are often accompanied by unintended decreases in essential mineral elements. Achieving a substantial reduction in grain Cd concentrations while maintaining appropriate levels of mineral elements therefore remains a major bottleneck in low-Cd rice breeding. Here, we report that pyramiding elite alleles of *OsNRAMP5* and *OsHMA3*, two key genes encoding Cd transporters, confers low Cd accumulation in grains without inducing sensitivity to manganese (Mn) deficiency in Layandabu (LAA), a tropical *japonica* rice cultivar. A serine-to-phenylalanine substitution at position 313 in OsNRAMP5^LAA^ weakens its interaction with OsVAP1-3, a vesicle-associated membrane protein (VAMP)-associated protein that facilitates OsNRAMP5 export from the endoplasmic reticulum (ER) to the plasma membrane. This weakened interaction results in partial retention of OsNRAMP5^LAA^ in the ER, thereby reducing Cd and Mn uptake. Introgression of the linked *OsNRAMP5*^*LAA*^ and *OsHMA3*^*LAA*^ alleles into the commercial rice cultivar Wushansimiao markedly reduced Cd concentrations in brown rice grown in Cd-contaminated fields without incurring a yield penalty, even under combined heat and low-Mn stress. Thus, our findings provide mechanistic insights into how low-Cd accumulation can be balanced with stress resilience and offer a promising strategy for developing rice cultivars with low-Cd grains and broad environmental adaptability.

## Teaser

This study demonstrates that the VAMP-associated protein OsVAP1-3 facilitates the export of OsNRAMP5 from the endoplasmic reticulum. The S313F substitution in OsNRAMP5^LAA^ weakens its interaction with OsVAP1-3 and causes partial retention of OsNRAMP5^LAA^ in the endoplasmic reticulum. Consequently reducing Cd and Mn uptake in rice. Importantly, pyramiding the weak allele *OsNRAMP5*^*LAA*^ and the strong allele *OsHMA3*^*LAA*^ substantially reduces grain Cd concentration without compromising Mn–superoxide dismutase (Mn-SOD) activity and stress tolerance.

## Introduction

Cadmium (Cd), a highly toxic heavy metal, is a group I carcinogen (Clemens and Ma, 2016; Wang et al., 2019). According to a recent survey of toxic metal contamination in global agricultural soils, Cd has the highest exceedance rate, affecting approximately 9% of surface soils (Hou et al., 2025). Rice (*Oryza sativa*) serves as a staple food for over half of the global population and is more prone to Cd accumulation than other cereal crops. It is also the primary dietary source of Cd intake (Zhao and Wang, 2020). Excessive Cd in rice grains therefore poses a serious threat to human health worldwide (Jing et al., 2023; Peera Sheikh Kulsum et al., 2023). Approximately 90% of global rice production occurs in Asia. Surveys of rice samples have shown that average Cd concentrations in rice grains are higher in Asia than in Europe, the Middle East, and North America (Jallad, 2015). Rice grains with excessive Cd have been reported in several Asian countries, including China, Japan, Bangladesh, India, and Sri Lanka (Shi et al., 2020). Currently, developing and cultivating low-Cd rice varieties represents a practical and cost-effective approach to mitigating Cd accumulation in rice grains and ensuring safe rice production.

Cd accumulation in rice grains occurs through two major pathways: (i) root uptake from the soil followed by transport to the grains during grain filling and (ii) remobilization of Cd accumulated in leaves and nodes and its subsequent translocation to the grains. Among these, the first pathway is the primary source of grain Cd when rice is grown in fields with clean air but Cd-contaminated soil (Huang et al., 2022). Therefore, limiting Cd uptake and root-to-shoot translocation is crucial for reducing grain Cd concentrations. Several genes are involved in Cd transport; among them, *OsNRAMP5* and *OsHMA3* are key genes associated with Cd uptake and root-to-shoot translocation, respectively (Nakanishi et al., 2006; Ueno et al., 2010; Ishikawa et al., 2012; Yan et al., 2019; Chang et al., 2020a; Tan et al., 2020a). OsNRAMP5 is localized to the distal side of the plasma membrane (PM) in exodermis and endodermis cells of the root and primarily mediates Cd and manganese (Mn) uptake (Ishikawa et al., 2012; Sasaki et al., 2012). Additionally, it facilitates Mn root-to-shoot translocation and unloading from the leaf sheath xylem (Yang et al., 2014; Huang et al., 2023). Knockout of *OsNRAMP5* markedly reduces Cd and Mn concentrations in shoots and grains under low and moderate Cd stress. Unexpectedly, we found that its knockout facilitates root-to-shoot Cd translocation, resulting in higher shoot Cd concentrations in rice plants exposed to high Cd stress (Tang et al., 2022). *OsHMA3* encodes a tonoplast transporter that sequesters Cd in vacuoles of root cells, thereby reducing root-to-shoot Cd translocation (Ueno et al., 2010). Seven nucleotide variations in the promoter region of *OsHMA3* enhance its expression levels, leading to significant differentiation between *indica* and *japonica* rice subspecies, which partially accounts for the divergence in Cd accumulation between these subspecies (Liu et al., 2020).

Knockout of *OsNRAMP5* markedly reduces the uptake of Cd and Mn in rice (Ishikawa et al., 2012; Sasaki et al., 2012). Mn is an indispensable micronutrient for plant growth, reproduction, and stress tolerance. It is involved in photosynthesis, scavenging of reactive oxygen species, and lignin biosynthesis and serves as a direct cofactor for more than 30 enzymes (Schmidt et al., 2016; Shao et al., 2017). In soils with moderate Cd contamination and abundant bioavailable Mn, knockout of *OsNRAMP5* reduced grain Cd concentrations by >90% without significant growth retardation or yield penalty (Ishikawa et al., 2012; Tang et al., 2017). This phenotype of *osnramp5* was attributed to leaf Mn concentrations remaining above the threshold required for normal growth and development (50–100 mg/kg dry weight [DW]) (Mitani-Ueno et al., 2018) when plants were grown in soils with sufficient Mn, despite a substantial decline in Mn uptake. Several *japonica* and *indica* rice cultivars harboring loss-of-function mutations in *OsNRAMP5* have been registered in Japan and China (Ishikawa, 2020; Tang et al., 2023; Wang et al., 2025). However, in paddy fields experiencing brown spot disease outbreaks, the cultivar Koshihikari Kan No. 1, which carries a frameshift mutation in *OsNRAMP5*, exhibited higher susceptibility to brown spot disease and lower yield than wild-type (WT) Koshihikari (Toshimitsu et al., 2017). Mn superoxide dismutase (Mn-SOD) plays an important role in rice thermotolerance by scavenging reactive oxygen species (Shiraya et al., 2015). Our previous study showed that *osnramp5* mutants in the backgrounds of the *indica* rice cultivar Huazhan (HZ) and the *japonica* rice cultivar Zhonghua 11 were sensitive to low-Mn stress and high temperature during the heading stage. Grain yields of *osnramp5* mutants were 30.6%–44.0% lower in low-Mn soils and 20.8%–31.2% lower under high-temperature conditions than those of the corresponding WT plants (Dong et al., 2021). Given the wide variation in Mn bioavailability among different soils and the ongoing trend of global warming, the risk of stress susceptibility and yield loss in *osnramp5* mutants will persist unless their Mn uptake is enhanced.

Breaking the trade-off between low Cd accumulation and stress resilience is currently an urgent and challenging task in low-Cd rice breeding. Two potential strategies to address this challenge include (i) altering the substrate selectivity of Cd transporters through specific amino acid substitutions and (ii) identifying favorable alleles of genes encoding Cd transporters. To date, no Cd-specific transporter has been identified in plants, and no study has reported the successful manipulation of a metal transporter to achieve an exclusive loss of Cd transport activity *in planta*. A substitution of glutamine (Q) with lysine (K) at position 337 in the OsNRAMP5 protein resulted in moderate uptake of Cd and Mn, higher tolerance to Mn deficiency than *osnramp5* seedlings in hydroponic assays, and approximately 50% lower grain Cd concentrations than the WT in paddy fields (Kuramata et al., 2022). However, the stress tolerance of the *OsNRAMP5*^*Q337K*^ mutant in paddy fields and the mechanism underlying its reduced Cd and Mn uptake remain unclear. In Pokkali, an ancient *indica* cultivar, tandem duplication of *OsNRAMP5* doubled its expression and enhanced Cd and Mn uptake while reducing root-to-shoot Cd translocation. Introgression of this allele into Koshihikari, a widely cultivated *japonica* rice variety, decreased brown rice Cd concentrations by 64% and increased straw Mn concentrations by 74% without compromising grain yield (Yu et al., 2022). Although this allele has a greater impact on reducing grain Cd accumulation, it remains unknown whether rice cultivars carrying this allele would exhibit symptoms of high Mn toxicity when grown in acidic soils with high Mn bioavailability. Therefore, the development of low-Cd rice cultivars requires identification of superior allelic variants of transporter genes that balance reduced Cd accumulation with stress resilience.

Leaf Mn concentrations are much higher than required in WT rice plants but are insufficient to withstand low-Mn stress in *osnramp5* mutants. In this study, we identified a low-Cd rice germplasm, Layandabu (LAA). Genetic analysis indicated that it carries a weak allele of *OsNRAMP5* and a strong allele of *OsHMA3*. Introgression of these two genetically linked alleles into a commercial rice cultivar resulted in low grain Cd concentrations under Cd-contaminated field conditions, without causing stress sensitivity due to Mn-deficiency. Furthermore, we demonstrated that OsVAP1-3, a vesicle-associated membrane protein (VAMP)-associated protein, binds to OsNRAMP5 in the endoplasmic reticulum (ER) and facilitates its export from the ER. A serine (S)-to-phenylalanine (F) substitution at position 313 in OsNRAMP5^LAA^ weakens its interaction with OsVAP1-3 and causes partial ER retention of OsNRAMP5^LAA^, consequently diminishing Cd and Mn uptake in rice. This work reveals a previously unidentified regulatory mechanism governing OsNRAMP5 intracellular trafficking and provides a proof-of-concept breeding strategy to break the trade-off between low-Cd accumulation and stress resilience.

## Results

### Rice cultivar LAA exhibits relatively low Cd and Mn accumulation without compromised tolerance to Mn deficiency

**Figure 1:**
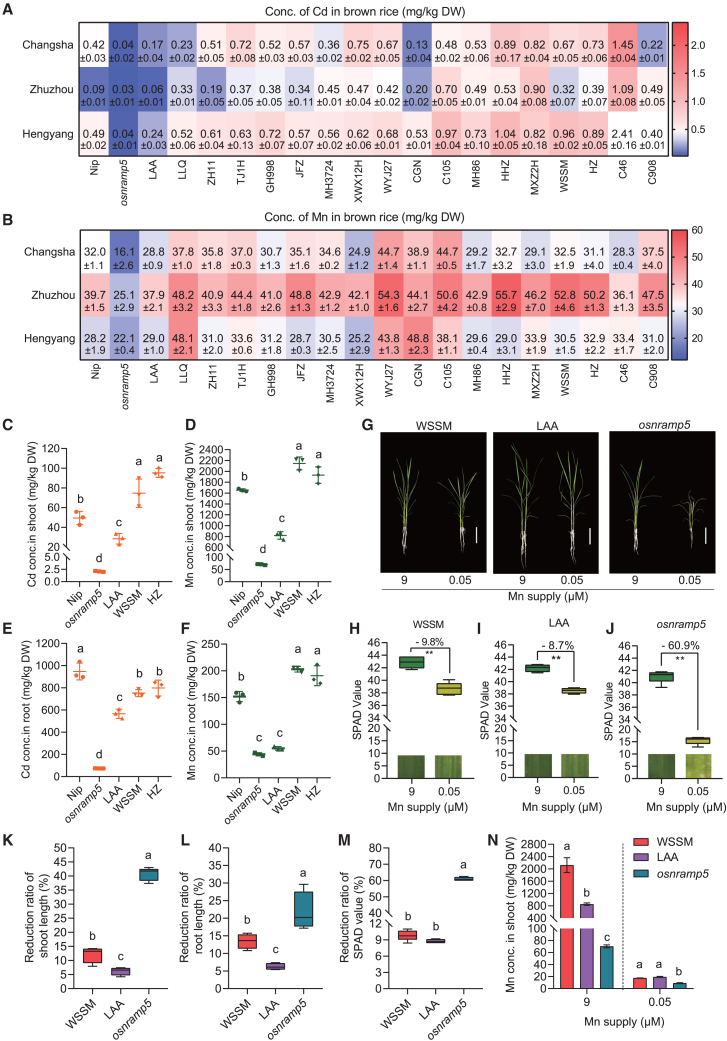
Morphological and physiological characteristics of the tropical *japonica* rice cultivar Layandabu (LAA). **(A and B)** Cd **(A)** and Mn **(B)** concentrations in brown rice of 20 relatively low-Cd and high-Cd rice cultivars grown in three paddy fields with soil total Cd concentrations of 0.9 (Changsha), 0.5 (Zhuzhou), and 1.1 (Hengyang) mg/kg, respectively. Conc., concentration; DW, dry weight. **(C–F)** Cd and Mn concentrations in the shoots **(C and D)** and roots **(E and F)** of Layandabu (LAA) and other rice cultivars grown in standard nutrient solution containing 9 μM Mn for 2 weeks followed by treatment with 0.5 μM Cd for 2 weeks. **(G–N)** Characteristics of LAA under low-Mn stress. LAA, WSSM, and *osnramp5* plants in the WSSM background were grown in standard nutrient solution containing 9 μM Mn for 30 days or in standard nutrient solution containing 9 μM Mn for 3 days and then transferred to nutrient solution with 0.05 μM Mn for an additional 27 days. Plants **(G)** and leaves **(H–J)** were photographed, and relative chlorophyll contents (SPAD values; **H–J**) and shoot Mn concentrations **(N)** were determined. Scale bar, 10 cm. **(K–M)** Plant tolerance to Mn depletion was evaluated by analyzing the extent of the decrease in shoot length **(K)**, root length **(L)**, and SPAD value **(M)** under 0.05 μM Mn relative to those under 9 μM Mn. Values are presented as means ± SD of three biological replicates in **(A)–(F) and (N)**. Box-and-whisker plots display minima and maxima, 25th and 75th percentiles (box), and medians (center line) of five biological replicates in **(H)**–**(M)**. Two asterisks indicate significant differences by Student’s *t-*test (*P* < 0.01). Different lowercase letters indicate significant differences based on one-way ANOVA followed by Tukey’s test (*P* < 0.05).

Grain Cd concentration is a quantitative trait influenced by both genetic and environmental factors. We screened 232 rice cultivars from a collection of representative hybrid parental lines in China (Lv et al., 2020), a core collection of Asian rice cultivars (Tan et al., 2020b), and the 3000 Rice Genomes Project (3K-RG) population (Wang et al., 2018) (Supplemental Table 1) using hydroponic experiments with Cd treatment. From this screening, we selected 20 rice cultivars with relatively low or high shoot Cd concentrations. To identify rice germplasm with consistently low grain Cd accumulation across diverse environments, we grew these 20 cultivars in three experimental paddy fields with different soil Cd concentrations. In these fields, grain Cd and Mn concentrations varied widely among cultivars, ranging from 0.025 to 2.407 mg/kg and from 16.1 to 55.7 mg/kg, respectively. *osnramp5* mutants in the background of Wushansimiao (WSSM), an elite *indica* rice cultivar, exhibited the lowest grain Cd and Mn concentrations. Brown rice Cd concentrations in LAA, a tropical *japonica* cultivar, ranged from 0.057 to 0.244 mg/kg; these values were only slightly higher than those in *osnramp5* but lower than those in the other 18 cultivars (Figure 1A). In contrast, brown rice Mn concentrations in LAA (28.8–37.9 mg/kg) were consistently higher than those in *osnramp5* across all tested fields (Figure 1B).

In hydroponic assays supplemented with 0.5 μM Cd and 9 μM Mn, Cd and Mn concentrations in both shoots and roots were lower in LAA than in Nipponbare (Nip), a *japonica* rice cultivar, and in WSSM and HZ, two *indica* rice cultivars. Shoot Cd and Mn concentrations and root Cd concentrations were higher in LAA than in *osnramp5* seedlings, whereas root Mn concentrations were similar between LAA and *osnramp5* (Figures 1C–1F). These findings suggest that LAA exhibits relatively low Cd and Mn accumulation. To determine whether LAA could maintain tolerance to Mn deficiency despite reduced Mn accumulation, we performed a hydroponic experiment under Mn-depleted conditions. *osnramp5* plants exhibited clear Mn-deficiency symptoms, including stunted growth and wilting of young leaves (Figure 1G). Plant height, root length, and chlorophyll relative content (soil and plant analyzer development, SPAD value) were lower in the Mn-depleted nutrient solution than in the standard nutrient solution. The corresponding reduction ratios in LAA were comparable to or lower than those in WSSM and were significantly greater in *osnramp5* (Figures 1G–1M; Supplemental Figures 1A–1C). These results indicate that the tolerance of LAA to Mn deficiency is comparable to that of WSSM but notably superior to that of *osnramp5*. Under a 9 μM Mn supply, shoot Mn concentration in LAA (∼854.4 mg/kg) was ∼40% of that in WSSM (∼2123.0 mg/kg) but 12-fold higher than that in *osnramp5* (∼70.3 mg/kg). Under a 0.05 μM Mn supply, shoot Mn concentration in LAA was similar to that in WSSM and twice that in *osnramp5* (∼9.1 mg/kg) (Figure 1N).

### Rice cultivar LAA harbors two low-Cd alleles of *OsHMA3* and *OsNRAMP5*

**Figure 2:**
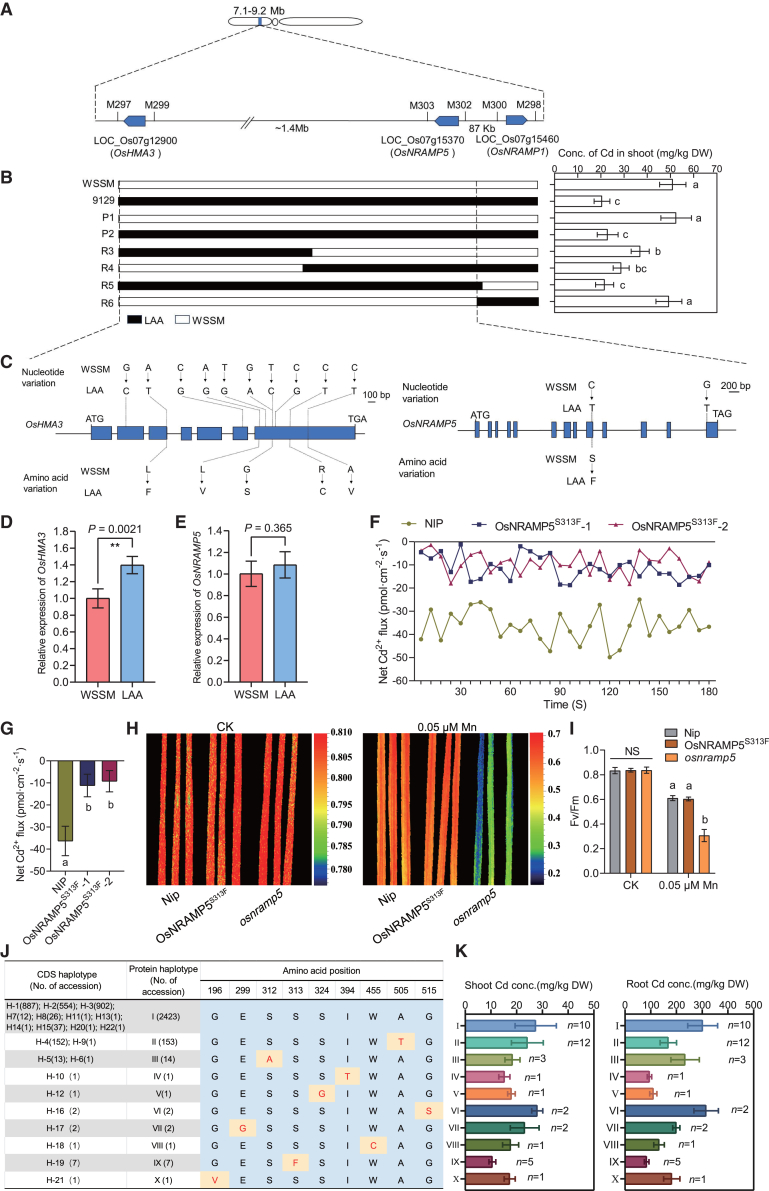
Map-based cloning and characterization of *OsNRAMP5*^*LAA*^ and *OsHMA3*^*LAA*^. **(A)** A QTL responsible for low Cd accumulation in LAA was mapped using an F_2_ population derived from a cross between LAA and WSSM. **(B)** Genotypes and shoot Cd concentrations of homozygous F_2:3_ recombinants. White and black bars represent LAA and WSSM homozygous genomic regions, respectively. M297–M303, KASP markers. **(C)** Genomic structures and sequence variations of the two candidate genes *OsNRAMP5* and *OsHMA3* in LAA and WSSM. Blue boxes, exons. **(D and E)** Expression levels of *OsHMA3***(D)** and *OsNRAMP5***(E)** in the roots of LAA and WSSM. **(F and G)** Net Cd^2+^ flux **(F)** and mean Cd^2+^ flux **(G)** in root microzones of OsNRAMP5^S313F^ and wild-type Nip seedlings exposed to Cd.. **(H and I)** Comparison of the maximum quantum efficiency of photosystem II (Fv/Fm) in OsNRAMP5^S313F^, *osnramp5*, and wild-type Nip seedlings under untreated control (CK) and Mn-deficiency (0.05 μM Mn) conditions. False-color images of Fv/Fm are shown in **(H),** and Fv/Fm values are quantitatively analyzed in **(I)**. **(J and K)** Haplotype analysis of the OsNRAMP5 protein **(J)** and shoot and root Cd concentrations in corresponding cultivars **(K)**. Red letters indicate variant amino acids in **(J)**. Rice cultivars harboring different OsNRAMP5 haplotypes were grown in standard nutrient solution for 2 weeks and then exposed to 0.5 μM Cd for an additional 7 days. Cd concentrations in shoots and roots were determined **(K)**. *n* denotes the number of representative rice accessions. Values are presented as means ± SD of three biological replicates. Two asterisks indicate significant differences by Student’s *t-*test (*P* < 0.01). Different lowercase letters indicate significant differences based on one-way ANOVA followed by Tukey’s test (*P* < 0.05). NS, not significant.

To identify the genetic basis of low Cd accumulation in LAA, we crossed LAA with WSSM and examined the phenotypes of 456 individuals from the segregating F_2_ population in a hydroponic experiment supplemented with 0.5 μM Cd. Shoot Cd concentrations in the F_2_ population showed a continuous distribution and varied considerably (Supplemental Figure 2A), suggesting that shoot Cd accumulation is controlled by quantitative trait loci (QTLs). We selected two pools of phenotypically extreme seedlings (low and high shoot Cd concentrations), along with their two parents, LAA and WSSM, for bulked segregant analysis (BSA) coupled with whole-genome sequencing. We identified a highly dense region of SNPs and insertions or deletions (indels) closely associated with variation in shoot Cd concentrations within the interval between 7.1 and 9.2 Mb on the short arm of chromosome 7 (Figure 2A; Supplemental Figure 2B). Three Cd transporter genes, *OsHMA3*, *OsNRAMP5*, and *OsNRAMP1*, were located in this interval and were identified as candidate genes. Therefore, we developed six KASP markers (M297–M303) flanking these genes to map the causal genes in this region (Supplemental Table 2). Using these markers, we isolated six plants with recombination between *OsNRAMP5* and *OsHMA3* from 226 F_2_ plants and four plants with recombination between *OsNRAMP5* and *OsNRAMP1* from 1257 F_2_ plants. Recombinant F_2_ plants were self-pollinated to produce F_2:3_ populations. Integrating the genotypes and shoot Cd concentrations of homozygous F_2:3_ progeny narrowed the QTL to a region between markers M297 and RM302 (Figure 2B), suggesting that *OsHMA3* and *OsNRAMP5*, but not *OsNRAMP1*, are the candidate genes underlying this QTL (Figure 2C).

A previous study reported that the rice cultivar PA64S, which harbors a superior allele of *OsHMA3,* exhibits higher *OsHMA3* expression levels in roots than the rice cultivar 93-11, resulting in lower Cd concentrations in PA64S shoots and grains (Liu et al., 2020). Sequencing of the coding sequences (CDSs) and 2 kb promoter regions of *OsHMA3* revealed that *OsHMA3*^*LAA*^ is identical to *OsHMA3*^*PA64S*^, whereas *OsHMA3*^*WSSM*^ is identical to *OsHMA3*^*93−11*^ (Figure 2C; Supplemental Figure 2C). Consistently, analysis of *OsHMA3* expression in roots showed higher transcript levels in LAA than in WSSM (Figure 2D).

Sequencing of the *OsNRAMP5* CDSs in LAA and WSSM revealed two SNPs. Of these, only one was a non-synonymous mutation: a C-to-T substitution at position 938 (from ATG) that results in an S-to-F substitution at position 313 in OsNRAMP5^LAA^ (Figure 2C). The expression level of *OsNRAMP5* did not differ significantly between the two cultivars (Figure 2E). To determine the distribution of the C938T SNP in the natural rice population, we analyzed variation in *OsNRAMP5* CDSs among 2605 rice cultivars using the 3K-RG database (Wang et al., 2018). The *OsNRAMP5* CDS was classified into 22 haplotypes. Among these, three dominant haplotypes (H1, H2, and H3) contained synonymous SNPs and together accounted for 90% of the variation (Supplemental Figures 3A and 3B). Most *japonica* cultivars belonged to H1, whereas most *indica* cultivars belonged to H2, H3, and H4 (Supplemental Figure 3B), indicating differentiation in *OsNRAMP5* CDSs between *indica* and *japonica* subspecies. The amino acid sequence of OsNRAMP5 was divided into 10 haplotypes, of which haplotype I accounted for 93% (Figure 2J), indicating that *OsNRAMP5* is highly conserved evolutionarily. Only seven rice cultivars carried haplotype IX of *OsNRAMP5*^*LAA*^ (Figure 2J), indicating that C938T is a rare allelic variant. Shoot and root Cd concentrations in haplotype IX cultivars were lower than those in cultivars carrying other haplotypes (Figure 2K). To verify whether *OsNRAMP5*^*C938T*^ contributes to reduced Mn and Cd uptake in LAA, we generated another F_2_ population by crossing the tropical *japonica* cultivar 11009 (carrying *OsNRAMP5*^*LAA*^) with the *indica* cultivar HZ (carrying *OsNRAMP5*^*WSSM*^). In frequency distribution histograms of shoot Cd and Mn concentrations in the F_2_ segregation population, the peaks corresponding to the TT, TC, and CC genotypes at position 938 of *OsNRAMP5* could be clearly distinguished (Supplemental Figures 3C and 3D). Scatter plots of the F_2_ population further showed that distinct genotypes at position 938 were associated with significantly different Cd and Mn concentrations in shoots (Supplemental Figures 3E and 3F). These findings confirm that genotypes at position 938 co-segregate with shoot Cd and Mn accumulation phenotypes, reflecting a genetic characteristic of incomplete dominance.

We used prime editing technology to mutate S^313^ of OsNRAMP5 to F^313^, creating OsNRAMP5^S313F^ plants in the Nip background (Supplemental Figure 4A). Two *osnramp5* lines with frameshift mutations of *OsNRAMP5* in the Nip background were generated as nonfunctional controls. Non-invasive micro-test technology (NMT) was used to monitor net Cd^2+^ flux in the root tip microenvironment. OsNRAMP5^S313F^ seedlings showed notably lower net Cd^2+^ flux than WT Nip plants (Figure 2F and 2G), suggesting that the S313F substitution in OsNRAMP5 reduces Cd uptake. Next, we analyzed plant height, root length, and the maximum quantum efficiency of photosystem II (PSII) in OsNRAMP5^S313F^, *osnramp5*, and WT plants grown in hydroponic solutions with or without Cd or Mn depletion. Knockout of *OsNRAMP5* in the Nip background inhibited seedling growth in the standard nutrient solution and reduced tolerance to low Mn stress but enhanced Cd tolerance (Figure 2H and 2I; Supplemental Figure 4B and 4C). Notably, the S313F substitution did not affect seedling growth or low-Mn tolerance but enhanced Cd tolerance (Figures 2H and 2I; Supplemental Figure 4B and 4C). Under 0.5 μM Cd treatment, OsNRAMP5^S313F^ plants showed shoot Cd and Mn concentrations and root Cd concentrations intermediate between those of Nip and *osnramp5*, whereas root Mn concentrations were as low as those in *osnramp5* (Supplemental Figures 4D and 4E). The root-to-shoot translocation ratios of Cd and Mn in OsNRAMP5^S313F^ plants were comparable to those of WT plants (Supplemental Figures 4F and 4G). S is a potentially phosphorylatable amino acid residue which is often mutated to alanine (A) or F to mimic a non-phosphorylatable form. To test whether reduced Cd and Mn accumulation in OsNRAMP5^S313F^ plants is related to dephosphorylation caused by the S313F substitution, we generated OsNRAMP5^S313A^ plants in the Nip background using prime editing (Supplemental Figure 4A). However, OsNRAMP5^S313A^ plants displayed only slightly lower root Mn concentrations than WT plants, and their Cd and Mn accumulation profiles were completely different from those of OsNRAMP5^S313F^ plants (Supplemental Figures 4D and 4E). Collectively, these results demonstrate that F^313^ is a functional variant residue in OsNRAMP5^LAA^ and that the phosphorylation status of S^313^ does not play a major role in regulating OsNRAMP5 function.

### *OsNRAMP5*^*LAA*^ reduces Cd uptake without compromising tolerance to combined heat and low-Mn stress

**Figure 3:**
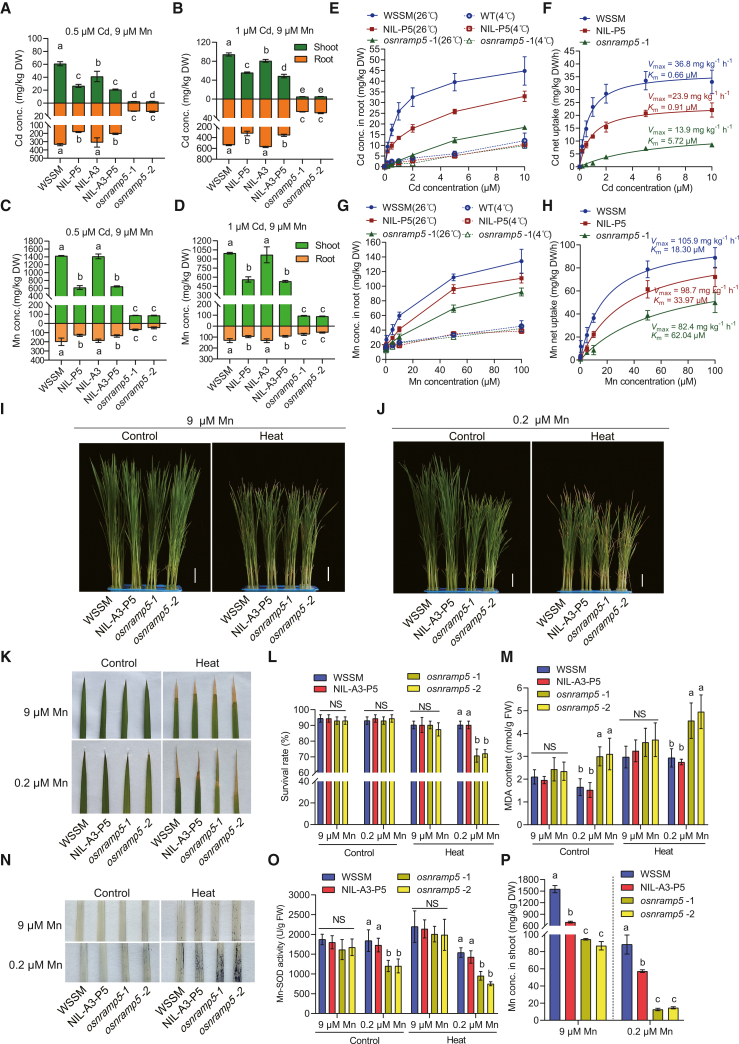
Comparison of Cd and Mn accumulation patterns and thermotolerance among NILs, *osnramp5,* and wild-type plants. **(A–D)** Cd **(A and B)** and Mn **(C and D)** concentrations in shoots and roots of WSSM (wild type), *osnramp5* mutant, NIL-*OsNRAMP5*^*LAA*^ (NIL-P5), NIL-*OsHMA3*^*LAA*^ (NIL-A3), and NIL-*OsHMA3*^*LAA*^-*OsNRAMP5*^*LAA*^ (NIL-A3-P5) grown in nutrient solution containing 9 μM Mn and 0.5 or 1 μM Cd for 14 days. **(E–H)** Kinetics of Cd and Mn uptake in NIL-P5, *osnramp5*, and WSSM plants. **(E andG)** Uptake of Cd **(E)** and Mn **(G)** at 26°C and 4°C. **(F andH)** Net uptake of Cd **(F)** and Mn **(H)**. Net uptake was calculated by subtracting apparent uptake at 4°C from that at 26°C, with *K*_m_ and *V*_max_ values shown next to the curves. **(I–P)** Thermotolerance assessment of WSSM, NIL-A3-P5, and *osnramp5* seedlings under 9 and 0.2 μM Mn. Two-week-old seedlings grown in nutrient solution containing 9 **(I)** or 0.2 μM Mn **(J)** were subjected to heat stress (45°C) for 3 days, followed by cultivation at 28°C for 7 days to allow recovery. Untreated controls consisted of 14-day-old seedlings cultured at 28°C for 10 days under the corresponding Mn concentrations. Plants were photographed **(I and J)**, and survival rates **(L)** were analyzed after 7 days of recovery. Scale bar, 3 cm. The third leaf blade was photographed **(K)** and stained with nitroblue tetrazolium (NBT) **(N)**. Malondialdehyde (MDA) content **(M)** and Mn concentration in shoots **(P)** were determined after exposure to heat stress for 3 days. Mn-SOD activity in shoots **(O)** was measured after 6 h of heat stress. Values are presented as means ± SD of three biological replicates in **(A)–(H) and (K)**–**(P)**. Different lowercase letters indicate significant differences based on one-way ANOVA followed by Tukey’s test (*P* < 0.05). NS, not significant.

To elucidate the genetic effects of *OsNRAMP5*^*LAA*^ and its application potential in low-Cd rice breeding, we generated three near-isogenic lines (NILs) via introgression of the *OsNRAMP5*^*LAA*^, *OsHMA3*^*LAA*^, and *OsHMA3*^*LAA*^-*OsNRAMP5*^*LAA*^ alleles from LAA into WSSM. Additionally, we produced two *osnramp5* lines with frameshift mutations in *OsNRAMP5* in the WSSM background using CRISPR–Cas9. Under standard nutrient solution conditions without stress treatment (Yoshida et al., 1976), growth did not differ significantly among NIL-*OsNRAMP5*^*LAA*^, *osnramp5*, and WSSM (Supplemental Figures 4A–4C). Upon exposure to 0.5 or 1 μM Cd in the presence of 9 μM Mn for 14 days, NIL-*OsNRAMP5*^*LAA*^ and *osnramp5* plants were taller than WSSM (Supplemental Figures 5A–5C), indicating that *OsNRAMP5*^*LAA*^ and *osnramp5* alleviate Cd-induced growth inhibition and enhance Cd tolerance. Under 0.5 μM Cd treatment, relative to WSSM, NIL-*OsNRAMP5*^*LAA*^ exhibited 56.3% and 45.3% lower Cd concentrations in shoots and roots, respectively, whereas the two *osnramp5* lines exhibited 95.8%–96.0% and 96.0%–96.2% lower Cd concentrations in shoots and roots, respectively (Figure 3A). Relative to WSSM, NIL-*OsNRAMP5*^*LAA*^ showed 56.5% and 35.0% lower Mn concentrations in shoots and roots, respectively, whereas the two *osnramp5* lines showed reductions of 93.8%–94.0% and 65.4%–75.9%, respectively (Figure 3C). Analysis of root-to-shoot Cd and Mn translocation revealed that the translocation ratios in NIL-*OsNRAMP5*^*LAA*^ were similar to those in WSSM, whereas *osnramp5* lines showed a higher Cd translocation ratio and a lower Mn translocation ratio (Supplemental Figures 5D and 5E), indicating that OsNRAMP5^LAA^ does not alter the efficiency of root-to-shoot Mn translocation.

To investigate the Cd and Mn uptake efficiency of OsNRAMP5^LAA^, we conducted short-term uptake experiments at 4°C and 26°C using WSSM, NIL-*OsNRAMP5*^*LAA*^, and *osnramp5* plants (Figures 3E and 3G). Uptake at 4°C reflects apoplastic adsorption of Cd or Mn. Net symplastic Cd or Mn uptake was estimated by subtracting uptake at 4°C from that at 26°C, and the resulting data were fitted to the Michaelis–Menten equation. The *V*_*max*_ values for Cd and Mn net uptake in NIL-*OsNRAMP5*^*LAA*^ were 65% and 93% of those in WSSM, respectively, and 1.7- and 1.2-fold those in *osnramp5*, respectively (Figures 3F and 3H). The *K*_*m*_ values for Cd and Mn net uptake in NIL-*OsNRAMP5*^*LAA*^ were 1.4- and 1.9-fold those in WSSM, respectively, and 16% and 55% of those in *osnramp5*, respectively (Figures 3F and 3H). These results suggest that *OsNRAMP5*^*LAA*^ is a weak allele, conferring Cd and Mn uptake capacities that are intermediate between those of *OsNRAMP5*^*WSSM*^ and *osnramp5*.

Under 0.25 or 0.05 μM Mn supply, *osnramp5* lines displayed hypersensitivity to low Mn, as indicated by retarded plant growth and chlorosis or wilting of young leaves (Supplemental Figures 6A–6C). By contrast, plant height and root length of NIL-*OsNRAMP5*^*LAA*^ were similar to those of WSSM (Supplemental Figures 6A–6C), suggesting that the low-Mn tolerance of NIL-*OsNRAMP5*^*LAA*^ is significantly superior to that of *osnramp5* and comparable to that of WSSM. Under 9 and 0.2 μM Mn supply, shoot Mn concentrations in NIL-*OsNRAMP5*^*LAA*^ were lower than those in WSSM but higher than those in *osnramp5* (Supplemental Figure 6D). Under 0.05 μM Mn supply, the shoot Mn concentration in NIL-*OsNRAMP5*^*LAA*^ was at the threshold for Mn-deficiency symptoms (10–20 mg/kg) (Shao et al., 2017) and similar to that in WSSM, whereas it was 1.9–2.4 times higher than that in *osnramp5* (7.5–9.0 mg/kg) (Supplemental Figure 6D), indicating that Mn uptake mediated by *OsNRAMP5*^*LAA*^ is sufficient to confer low-Mn tolerance.

Under 0.5 and 1 μM Cd treatment, we observed no significant differences in Cd tolerance, root Cd concentrations, or shoot and root Mn concentrations between NIL-*OsHMA3*^*LAA*^ and WSSM (Figures 3A–3D; Supplemental Figures 5A–5C). Under 0.5 μM Cd treatment, NIL-*OsHMA3*^*LAA*^ showed a 32.3% lower shoot Cd concentration and 16.5% lower root-to-shoot Cd translocation than WSSM (Figures 3A and 3C; Supplemental Figure 5D). Thus, *OsHMA3*^*LAA*^ represents a strong allele of *OsHMA3* associated with enhanced Cd sequestration in vacuoles of root cells compared with *OsHMA3*^*WSSM*^, consistent with previous reports (Liu et al., 2020). Under 1 μM Cd treatment, shoot Cd concentration was lower in NIL*-OsHMA3*^*LAA*^*-OsNRAMP5*^*LAA*^ (NIL-A3-P5) than in NIL-*OsNRAMP5*^*LAA*^ (Figure 3B), indicating that pyramiding these two low-Cd alleles exerts additive effects in reducing shoot Cd concentration under high Cd stress.

Because Mn-SOD plays a role in the rice heat response by scavenging superoxide radicals (O_2_^−^)^,^ we subjected 2-week-old seedlings of WSSM, NIL-A3-P5, and *osnramp5* to a 45°C heat treatment to compare their thermotolerance under normal Mn supply (9 μM) and low Mn supply (0.2 μM). Under 9 μM Mn, growth phenotypes, survival rates, and leaf tip wilting after heat treatment were comparable among the genotypes (Figures 3I–3L). Under 0.2 μM Mn, the survival rate of NIL-A3-P5 was equivalent to that of WSSM and significantly higher than that of *osnramp5*, whereas leaf tip wilting in NIL-A3-P5 was similar to that in WSSM but noticeably less severe than that in *osnramp5* after heat treatment (Figures 3I–3L). Because nitroblue tetrazolium (NBT) staining indicates O_2_^−^ accumulation, we performed NBT staining on leaves. Blue staining spots were denser in *osnramp5* leaves than in WSSM and NIL-A3-P5 leaves under 0.2 μM Mn after heat treatment (Figure 3N). Malondialdehyde (MDA) is a product of cell membrane oxidative damage. Under 9 μM Mn, we observed no significant difference in shoot MDA content or Mn-SOD activity among these plants under either control or heat treatment conditions (Figures 3M and 3O). In contrast, under 0.2 μM Mn, under both control and heat conditions, shoot MDA content and Mn-SOD activity in NIL-A3-P5 were comparable to those in WSSM, whereas *osnramp5* exhibited significantly higher MDA content and lower Mn-SOD activity than WSSM and NIL-A3-P5 (Figures 3M and 3O). Under 0.2 μM Mn, shoot Mn concentrations in *osnramp5* lines ranged from 11.3 to 15.7 mg/kg, which is close to the threshold for Mn-deficiency symptoms. In the other cases, shoot Mn concentrations of WSSM, NIL-A3-P5, and *osnramp5* plants were higher than or within the range of 50–100 mg/kg. Collectively, these results demonstrate that OsNRAMP5^LAA^ maintains shoot Mn concentrations and Mn-SOD activity sufficient to support thermotolerance comparable to that of WSSM under low-Mn conditions. By contrast, knockout of *OsNRAMP5* results in severely reduced shoot Mn concentrations and Mn-SOD activity, thereby compromising thermotolerance under low-Mn conditions.

### The S313F substitution inhibits OsNRAMP5 exit from the ER

**Figure 4:**
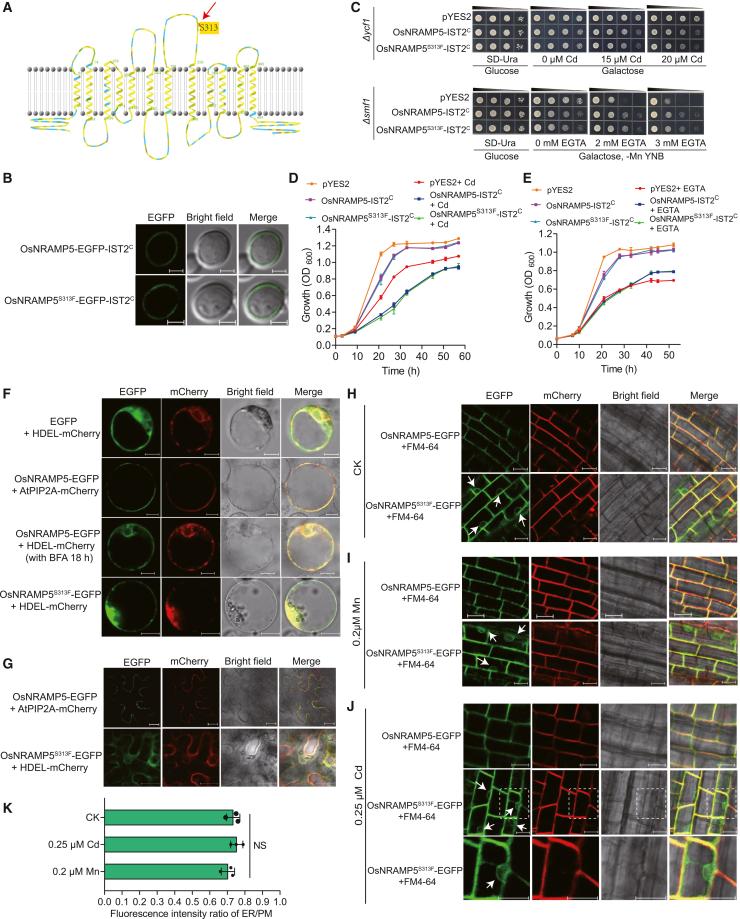
The S313F substitution causes partial retention of OsNRAMP5 in the endoplasmic reticulum. **(A)** Proposed topology of OsNRAMP5. The S313 residue is indicated by a red arrow. **(B)** Subcellular localization of OsNRAMP5-EGFP-IST2^C^ and OsNRAMP5^S313F^-EGFP-IST2^C^ in yeast cells. Scale bar, 2 μm. **(C)** Transport activity of OsNRAMP5 and OsNRAMP5^S313F^ in the yeast strains *Δycf1* (Cd-sensitive mutant) and *Δsmf1* (mutant defective in Mn uptake), respectively. Yeast cells carrying the empty vector pYES2 or different alleles of *OsNRAMP5* fused with *IST2*^*C*^ were grown on normal medium (SD–Ura) and treatment medium (SD–Ura containing 15 or 20 μM Cd, or 2 or 3 mM EGTA) for 3 days. A 10-fold dilution series of yeast cells is shown from left to right. **(D and E)** Growth curves of yeast cells expressing the empty vector pYES2 or different alleles of *OsNRAMP5* fused with *IST2*^*C*^ in liquid SD–Ura medium with or without 30 μM Cd **(D)** or 12.5 mM EGTA **(E)**. Values are presented as means ± SD of three biological replicates. **(F)** Subcellular localization of the OsNRAMP5-EGFP and OsNRAMP5^S313F^-EGFP fusion proteins in rice protoplasts with or without brefeldin A (BFA) treatment for 18 h. AtPIP2A-mCherry and HDEL-mCherry were used as plasma membrane (PM) and endoplasmic reticulum (ER) markers, respectively. Scale bar, 10 μm. **(G)** Subcellular localization of OsNRAMP5-EGFP and OsNRAMP5^S313F^-EGFP transiently expressed in tobacco epidermal cells. Scale bar, 20 μm. **(H–K)** Subcellular localization of OsNRAMP5-GFP variants in root cells of *proUbi:OsNRAMP5-EGFP*/*osnramp5* and *proUbi:OsNRAMP5*^*S313F*^*-EGFP*/*osnramp5* plants grown in nutrient solution containing 9 μM Mn (CK) **(H),** 0.2 μM Mn **(I),** or 0.25 μM Cd **(J)**. The PM (red fluorescence) was visualized by FM4-64 staining. The region indicated by the white dashed rectangle is enlarged in the lower panels of **(J)**. White arrows indicate OsNRAMP5^S313F^ localization in the perinuclear ER. Scale bar, 20 μm. **(K)** Ratio of EGFP fluorescence intensity at the ER to that at the PM in root cells of *proUbi:OsNRAMP5*^*S313F*^*-EGFP*/*osnramp5* plants under the treatments shown in **(H)–(J)**. NS, no significant difference by one-way ANOVA with Tukey’s test. Fluorescence signals were detected by confocal microscopy, and more than 12 images were analyzed for each assay **(F–J)**.

The predicted OsNRAMP5 secondary structure indicates that the S^313^ residue is located in the loop connecting the seventh and eighth transmembrane domains rather than within a transmembrane domain (Figure 4A), suggesting that S^313^ does not directly participate in Cd or Mn transmembrane transport. Alignment of the deduced amino acid sequence of this loop with homologous sequences showed that S^313^ is highly conserved among OsNRAMP5-like proteins (Supplemental Figure 7A). We expressed OsNRAMP5 and OsNRAMP5^S313F^ in the Cd-sensitive yeast strain *Δycf1* and the Mn-uptake-deficient yeast strain *Δsmf1* and compared their Cd and Mn transport activities. Growth phenotypes revealed that yeast expressing OsNRAMP5^S313F^ displayed higher Cd sensitivity and greater tolerance to low Mn than yeast expressing OsNRAMP5 (Supplemental Figure 7B). These phenotypes imply that OsNRAMP5^S313F^ has greater Cd and Mn transport capacity than OsNRAMP5 in the yeast system. To investigate the apparent discrepancy between the phenotypes of OsNRAMP5^S313F^ between rice and yeast, we expressed OsNRAMP5 and OsNRAMP5^S313F^ fused to enhanced green fluorescent protein (EGFP) at the C-terminus in yeast. Both fusion proteins localized to endomembrane compartments, with OsNRAMP5^S313F^ showing pronounced accumulation in the perinuclear ER (Supplemental Figure 7C). Possibly due to enhanced Cd and Mn transport into the perinuclear ER lumen—a site of active protein synthesis—yeast cells expressing OsNRAMP5^S313F^ were more sensitive to Cd and more tolerant to low-Mn stress. Because OsNRAMP5 is a PM-localized transporter in rice, we fused the IST2^C^ sequence (Jüschke et al., 2005) to the C-termini of OsNRAMP5 and OsNRAMP5^S313F^ to target them to the PM of yeast cells (Figure 4B). Spot dilution and growth curve assays revealed that yeast expressing OsNRAMP5-IST2^C^ and OsNRAMP5^S313F^-IST2^C^ exhibited similar growth under Cd supplementation or Mn deficiency (Figures 4C–4E), indicating that the S313F substitution does not affect the Cd and Mn transport activity of PM-localized OsNRAMP5.

Intracellular trafficking of metal transporters is mediated by vesicles and is essential for maintaining nutrient homeostasis in plants. In plant cells, transporters are usually synthesized in the ER, where they undergo proper folding and modification. Subsequently, correctly folded proteins are packaged into vesicles that bud from ER exit sites and are trafficked to their destinations via Golgi-dependent or Golgi-independent pathways (Gao and Chao, 2022). Confocal microscopy showed that OsNRAMP5-EGFP localized to the PM when expressed in rice protoplasts (Figure 4F), as reported previously (Sasaki et al., 2012). After treatment with brefeldin A, an ER-to-Golgi trafficking inhibitor, for 18 h, most OsNRAMP5-EGFP colocalized with the mCherry-fused ER marker protein, indicating retention of OsNRAMP5 in the ER (Figure 4F). This finding demonstrates that most OsNRAMP5 is trafficked to the PM via a Golgi-dependent pathway. Similarly, OsNRAMP5^S313F^-EGFP partially colocalized with the mCherry-fused ER marker protein, indicating ER localization in rice protoplasts (Figure 4F). These observations were confirmed using a tobacco transient expression system, in which OsNRAMP5 localized to the PM, whereas OsNRAMP5^S313F^ exhibited ER retention (Figure 4G).

Next, we overexpressed *OsNRAMP5-EGFP* and *OsNRAMP5*^*S313F*^*-EGFP* driven by the maize *Ubiquitin* promoter in Luohong3B (LH3B), an *indica* variety that lacks the 408 kb DNA segment containing *OsNRAMP5* due to mutagenesis (Lv et al., 2020) (Supplemental Figures 8A and 8B). Cd and Mn accumulation in plants overexpressing *OsNRAMP5-EGFP* was higher than that in LH3B (Supplemental Figures 8C and 8D), indicating that the *OsNRAMP5-EGFP* expression cassette functioned properly and restored Cd and Mn accumulation in these transgenic plants. Cd and Mn accumulation in plants overexpressing *OsNRAMP5*^*S313F*^*-EGFP* was lower than that in plants overexpressing *OsNRAMP5-EGFP* (Supplemental Figures 8C and 8D), confirming that the Cd and Mn uptake capacities of OsNRAMP5^S313F^ were lower than those of OsNRAMP5. Root cells of these overexpression plants grown in standard nutrient solution containing 9 μM Mn, 0.2 μM Mn, or 0.25 μM Cd were examined by confocal microscopy. When root cells were stained with FM4-64 to visualize the PM in red, OsNRAMP5-EGFP fluorescence signals completely overlapped with the red PM signal, whereas OsNRAMP5^S313F^-EGFP fluorescence signals partially overlapped with the PM signal and were also detected in the perinuclear ER (Figures 4H–4J), indicating that OsNRAMP5^S313F^ localizes to both the PM and ER. We further quantified EGFP fluorescence intensity in different subcellular compartments in plants overexpressing *OsNRAMP5*^*S313F*^*-EGFP*. The fluorescence intensity of OsNRAMP5^S313F^-EGFP in the ER was 69%–79% of that in the PM under different treatments (Figure 4K), indicating that OsNRAMP5^S313F^ protein abundance was slightly higher at the PM than in the ER. Collectively, these results demonstrate that the S313F variant impairs ER exit of OsNRAMP5, causing partial retention of OsNRAMP5 in the ER and thereby restricting Cd and Mn uptake in rice.

### OsVAP1-3 facilitates OsNRAMP5 export from the ER

**Figure 5:**
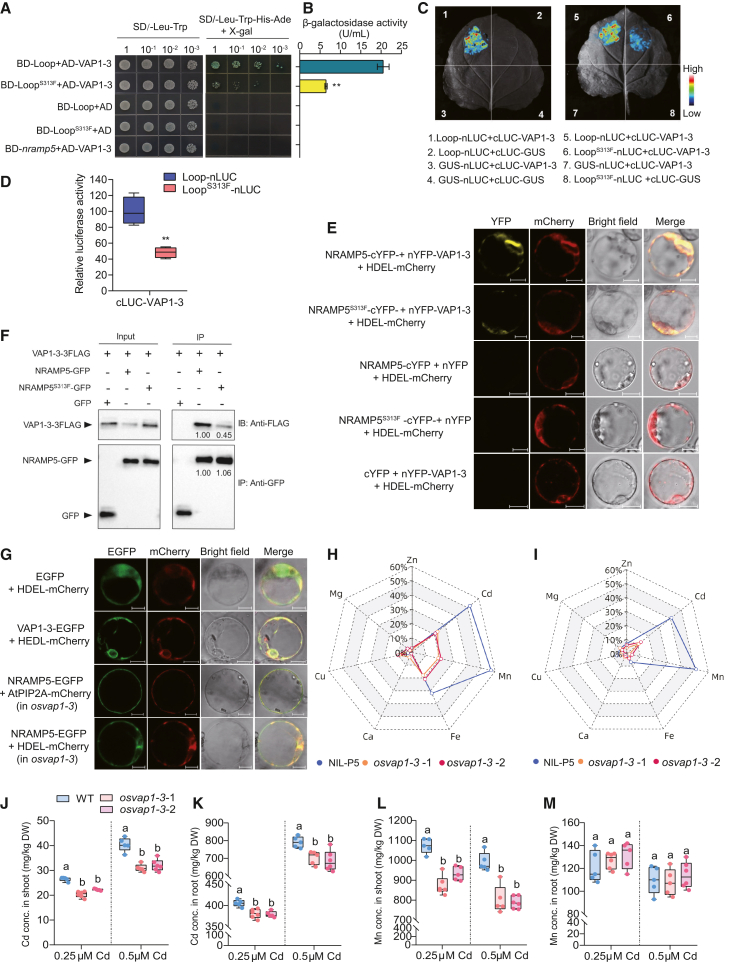
OsVAP1-3 interacts with OsNRAMP5 to modulate Cd and Mn accumulation in rice. **(A)** Split-ubiquitin membrane-based Y2H assay showing the interaction between Loop or Loop^S313F^ and OsVAP1-3. Yeast transformants were serially diluted and grown on SD (−Leu/Trp) and SD (−Leu/Trp/His/Ade/+X-gal) media. AD, prey vector; BD, bait vector. **(B)** β-Galactosidase activity assay quantifying interaction strength in **(A)**. Values represent means ± SD of three biological replicates. ∗∗indicates significant differences by Student’s *t-*test (*P* < 0.01). **(C)** Split-luciferase (split-LUC) assay examining the interaction between Loop or Loop^S313F^ and OsVAP1-3 in tobacco leaves. The color bar indicates LUC signal intensity. **(D)** Quantification of LUC activity in **(C)**. Box-and-whisker plots show minima and maxima, the 25th and 75th percentiles (box), and medians (center line) from 10 biological replicates. ∗∗ indicates significant differences by Student’s *t-*test (*P* < 0.01). **(E)** BiFC assay showing the interaction between OsNRAMP5 or OsNRAMP5^S313F^ and OsVAP1-3 in rice protoplasts. ER localization is indicated by the ER marker HDEL-mCherry. Scale bar, 10 μm. **(F)** Co-immunoprecipitation (Co-IP) assay demonstrating the interaction between OsNRAMP5-GFP or OsNRAMP5^S313F^-GFP and OsVAP1-3-3FLAG in rice protoplasts. ER proteins were extracted, immunoprecipitated using anti-GFP magnetic beads, and detected by western blotting with anti-FLAG or anti-GFP antibodies. GFP coexpressed with OsVAP1-3-3FLAG serves as a negative control. Immunoprecipitated OsNRAMP5-GFP or OsNRAMP5^S313F^-GFP was used as a loading control. Numbers indicate relative protein abundance. **(G)** Subcellular localization of OsVAP1-3-EGFP and OsNRAMP5-EGFP fusion proteins. Top images show the localization of EGFP (empty vector) and HDEL-mCherry as controls. The second row shows the localization of OsVAP1-3-EGFP together with HDEL-mCherry. The third and fourth rows show distinct localization patterns of OsNRAMP5-EGFP coexpressed with HDEL-mCherry and AtPIP2A-mCherry, respectively, in *osvap1-3* protoplasts. Scale bar, 10 μm. **(H and I)** Percentage decreases in the concentrations of seven divalent metal cations in shoots **(H)** and roots **(I)** of NIL-*OsNRAMP5*^*LAA*^ (NIL-P5) and two *osvap1-3* lines compared with the wild-type (WT) control. Values are presented as radar charts. **(J–M)** Cd **(J and K)** and Mn **(L and M)** concentrations in shoots and roots of *osvap1-3* lines and WT plants after treatment with 0.25 or 0.5 μM Cd for 2 weeks. Box-and-whisker plots show minima and maxima, the 25th and 75th percentiles (box), and medians (center line) from five to six biological replicates in **(J)**–**(M)**. Different lowercase letters indicate significant differences based on one-way ANOVA followed by Tukey’s test (*P* < 0.05).

To investigate how the S313F substitution causes partial retention of OsNRAMP5 in the ER, we screened a rice root cDNA library for proteins interacting with the non-transmembrane loop region (Loop) of OsNRAMP5, which contains S^313^, using a split-ubiquitin membrane-based yeast two-hybrid (Y2H) system. Protein export from the ER is mediated by vesicles (Nufer et al., 2002). Through this screen, we identified a putative VAMP-associated protein, OsVAP1-3. In Y2H assays, the interaction between OsVAP1-3 and the Loop was unaffected by low-Mn stress but was slightly enhanced by Cd stress (Supplemental Figure 9). Notably, S313F weakened the interaction between the Loop and OsVAP1-3 regardless of Mn deficiency or Cd stress (Figures 5A and 5B; Supplemental Figure 9). We next examined whether the OsNRAMP5 loop interacts with OsVAP1-3 *in planta* using a split-luciferase (LUC) complementation assay in *Nicotiana benthamiana* leaves. Coexpression of Loop–N-terminal luciferase fragment (nLUC) and C-terminal luciferase fragment (cLUC)–OsVAP1-3 yielded stronger LUC activity than coexpression of Loop^S313F^–nLUC and cLUC–OsVAP1-3 (Figures 5C and 5D), confirming that the interaction between the Loop and OsVAP1-3 is stronger than that between Loop^S313F^ and OsVAP1-3. Furthermore, a bimolecular fluorescence complementation (BiFC) assay in rice protoplasts confirmed that both OsNRAMP5 and OsNRAMP5^S313F^ interact with OsVAP1-3 (Figure 5E). Yellow fluorescent protein (YFP) signals completely overlapped with those of the ER marker (Figure 5E), indicating that these interactions occur in the ER. We further performed a co-immunoprecipitation assay using rice protoplasts. Anti-GFP beads co-immunoprecipitated more OsVAP1-3–FLAG fusion protein with OsNRAMP5-GFP than with OsNRAMP5^S313F^-GFP, but not with the GFP empty control (Figure 5F). Collectively, these results demonstrate that OsVAP1-3 interacts with OsNRAMP5 in the ER and that the S313F substitution weakens this interaction.

*OsVAP1-3* encodes a 237-amino acid protein containing a cytosolic major sperm domain, a coiled-coil domain, and a C-terminal transmembrane domain that anchors it to the membrane (Supplemental Figure 10A). The rice VAP family contains 19 members (Supplemental Figure 10B). VAP proteins are involved in intracellular trafficking of lipids and membrane proteins, linking the ER with various organelles such as mitochondria, chloroplasts, and the PM (Prosser et al., 2008; James and Kehlenbach, 2021; Li et al., 2022a; Man et al., 2024; Renna et al., 2024). OsVAP1-3 localized to the ER, as evidenced by its colocalization with an ER marker protein (Figure 5G). Quantitative RT–PCR revealed *OsVAP1-3* expression in various rice tissues throughout the growth period (Supplemental Figure 11A). To investigate the physiological function of *OsVAP1-3*, we generated *osvap1-3* mutants in the WSSM background using CRISPR–Cas9 technology (Supplemental Figure 11B). We then examined the effect of *OsVAP1-3* on OsNRAMP5 subcellular localization by transiently expressing the OsNRAMP5-EGFP fusion protein in protoplasts of *osvap1-3* mutants and WT plants. OsNRAMP5 predominantly localized to the PM of WT protoplasts, whereas approximately 48% of *osvap1-3* protoplasts displayed partial ER retention of OsNRAMP5 (Figure 5G; Supplemental Figure 11C). This cytological evidence indicates that *OsVAP1-3* positively regulates the efficiency of OsNRAMP5 export from the ER.

Under hydroponic conditions with or without 0.25 μM Cd, *osvap1-3* seedling growth was similar to that of the WT. After 14 days of exposure to 0.5 μM Cd, *osvap1-3* plants were slightly taller than WT plants (Supplemental Figures 11D–11F). Both NIL-*OsNRAMP5*^*LAA*^ and *osvap1-3* plants exhibited lower shoot Cd, Mn, and Fe concentrations than WT plants (Figures 5H, 5J, and 5M; Supplemental Figure 11G); however, the decrease in *osvap1-3* was less pronounced (Figure 5H). No significant differences were observed in shoot Mg, Zn, or Ca concentrations (Figure 5H). Additionally, shoot Cu concentrations were slightly lower in *osvap1-3* than in WT plants (Supplemental Figure 11H). Analysis of root concentrations of these seven divalent cations demonstrated that NIL-*OsNRAMP5*^*LAA*^ showed significant decreases only in Cd and Mn concentrations, whereas *osvap1-3* exhibited significant decreases only in Cd concentrations compared with the WT (Figure 5I). Similarly, upon exposure to 0.25 μM Cd, *osvap1-3* plants exhibited lower concentrations of Cd, Mn, Fe, and Cu in shoots and lower root Cd concentrations than WT plants (Figures 5J–5M; Supplemental Figures 11G and 11H). Previous studies have shown that OsNRAMP5 mainly mediates Cd and Mn uptake and is involved in additional Fe uptake (Sasaki et al., 2012). In our experiments, knockout of *OsVAP1-3* reduced Cd, Mn, and Fe accumulation, revealing that *OsVAP1-3* influences the uptake of these metals. Collectively, these results demonstrate that OsVAP1-3 facilitates the ER export and uptake function of OsNRAMP5.

The decrease in Cd and Mn accumulation caused by loss of *OsVAP1-3* function was smaller than that caused by the S313F substitution in OsNRAMP5 (Figures 5H and 5I). To explore the underlying mechanism, we examined interactions between OsNRAMP5 and seven other OsVAP family members with relatively high sequence similarity to OsVAP1*-*3. In the Y2H system, five of these OsVAP proteins also interacted with OsNRAMP5 (Supplemental Figure 12A). Further analysis demonstrated that all five OsVAP family members interacted with the Loop, and the S313F substitution in OsNRAMP5 weakened all of these interactions (Supplemental Figure 12B). These results suggest that (i) OsVAP family members may function redundantly in the ER export of OsNRAMP5, such that OsVAP1-3 function in *osvap1-3* mutants is partially compensated by other OsVAPs, and (ii) the S313F substitution weakens interactions between OsNRAMP5 and multiple OsVAP family members, including OsVAP1-3, resulting in a stronger genetic effect than the loss-of-function mutation of *OsVAP1-3*.

### Pyramiding *OsNRAMP5*^*LAA*^ and *OsHMA3*^*LAA*^ breaks the trade-off between low Cd accumulation and stress tolerance

**Figure 6:**
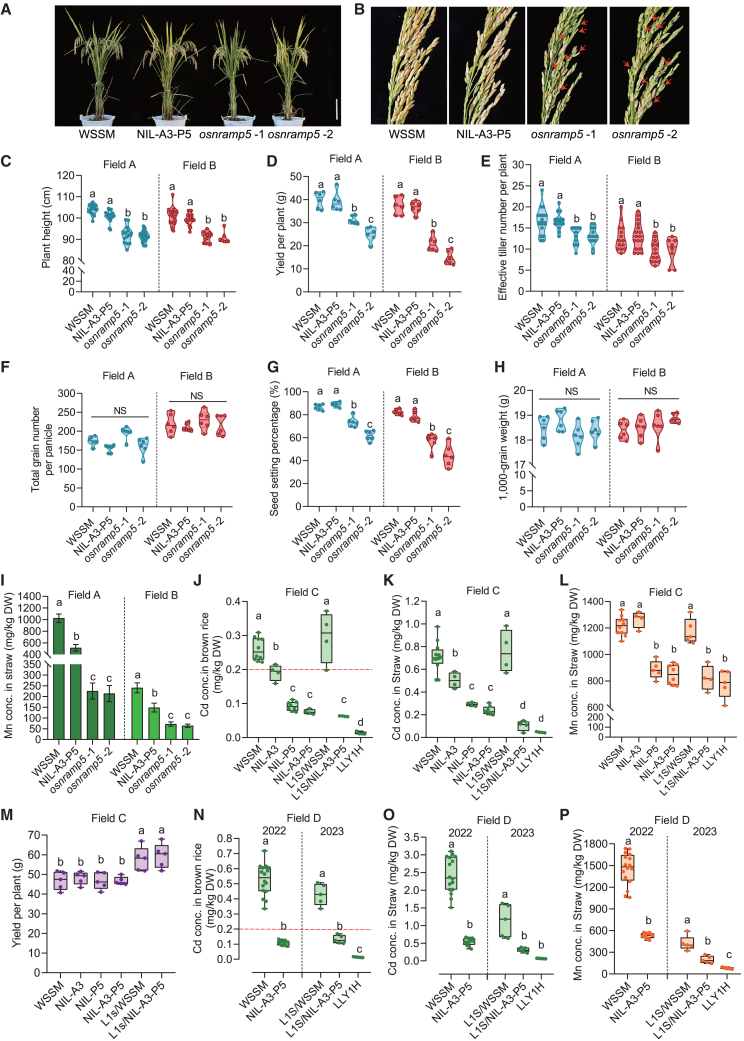
Comparison of agronomic traits and metal concentrations among WSSM, NILs, *osnramp5*, and their hybrid rice plants grown in paddy fields. **(A–I)** Morphological and physiological characteristics of NIL-*OsHMA3*^*LAA*^-*OsNRAMP5*^*LAA*^ (NIL-A3-P5), WSSM, and *osnramp5* cultivated in fields with soil total Mn concentrations of 312 mg/kg (Field A) and 102 mg/kg (Field B), respectively. Plants experienced high temperatures during the booting and heading stages. Plant morphology **(A)**, panicle morphology **(B)**, agronomic traits **(C–H),** and straw Mn concentrations **(I)** were assessed at maturity. Scale bar, 20 cm. Red arrows indicate open glumes. Horizontal bars within violin plots indicate the 25th percentile, median, and 75th percentile based on 6–16 biological replicates in **(C)–(H)**. Values in **(I)** are presented as means ± SD of four biological replicates. **(J–P)** Yield performance and metal accumulation characteristics of NIL-*OsNRAMP5*^*LAA*^ (NIL-P5), NIL-*OsHMA3*^*LAA*^ (NIL-A3), NIL-A3-P5, and their hybrid rice plants cultivated in fields with soil total Cd concentrations of 0.5 mg/kg (Field C) and 0.9 mg/kg (Field D). Hybrid rice plants L1S/WSSM, L1S/NIL-A3-P5, and LLY1H were generated by crossing L1S, an *osnramp5* sterile line, with WSSM, NIL-A3-P5, and *osnramp5*, respectively. Yield per plant **(M)**, brown rice Cd concentrations **(J and N)**, straw Cd concentrations **(K and O),** and straw Mn concentrations **(L and P)** were measured at maturity. Box-and-whisker plots show minima and maxima, the 25th and 75th percentiles (box), and medians (center line) from 4–16 biological replicates in **(J)–(P)**. Different lowercase letters indicate significant differences based on one-way ANOVA followed by Tukey’s test (*P* < 0.05).

In 2022, NIL-A3-P5, *osnramp5*, and WSSM (WT) were grown in Field A, which had abundant soil Mn (total Mn, 312 mg/kg; pH 6.6), and Field B, which had low soil Mn (total Mn, 102 mg/kg; pH 6.5). During the booting and heading stages, all plants experienced sustained high temperatures exceeding 38°C (Supplemental Figure 13A). In both fields, yield per plant and yield components of NIL-A3-P5 were comparable to those of WSSM (Figures 6A–6H). By contrast, yield per plant of *osnramp5* was 22.1%–37.5% lower than that of WSSM in Field A and 43.8%–60.3% lower in Field B (Figures 6A and 6D). *osnramp5* exhibited significantly lower plant height, seed-setting rates, and effective tiller numbers per plant than WSSM, resulting in significantly lower yields (Figures 6C–6H). Incomplete glume closure was observed in *osnramp5* panicles, whereas this phenotype was not detected in WSSM or NIL-A3-P5 (Figure 6B). In Field A, straw Mn concentrations in NIL-A3-P5 (∼514.7 mg/kg) were 50.3% of those in WSSM (∼1023.8 mg/kg) but 2.3–2.4 times those in *osnramp5* (213.8–225.3 mg/kg) (Figure 6I). In Field B, straw Mn concentrations in NIL-A3-P5 (∼148.6 mg/kg) were 61.8% of those in WSSM (∼240.5 mg/kg) but 2.1–2.3 times those in *osnramp5* (64.1–71.9 mg/kg) (Figure 6I). Brown rice Mn concentrations in NIL-A3-P5 were lower than or comparable to those in WSSM but 1.3–1.5 times those in *osnramp5* (Supplemental Figure 13B). Because *OsHMA3* does not affect Mn accumulation in rice, these results indicate that Mn uptake mediated by *OsNRAMP5*^*LAA*^ was sufficient to maintain tolerance to combined heat and low-Mn stress in paddy fields.

Because WSSM is an elite male parent for hybrid rice, we crossed WSSM and NIL-A3-P5 with Lian1S (L1S), an *osnramp5* mutant female parent, to generate the hybrid rice varieties L1S/WSSM and L1S/NIL-A3-P5, respectively. Crossing L1S with *osnramp5* in another genetic background generated the hybrid rice variety LLY1H, which serves as a low-Cd control. In 2022 and 2023, NILs and their hybrid rice offspring were grown in Fields C and D, which had soil total Cd concentrations of 0.5 mg/kg (pH 5.6) and 0.9 mg/kg (pH 5.8), respectively. In Field C, relative to WSSM, Cd concentrations in straw and brown rice were 29.1% and 26.6% lower in NIL-*OsHMA3*^*LAA*^, respectively; 58.9% and 64.4% lower in NIL-*OsNRAMP5*^*LAA*^, respectively; 66.6% and 71.0% lower in NIL-A3-P5, respectively; and 85.4% and 75.8% lower in L1S/NIL-A3-P5, respectively (Figures 6J and 6K). In Field D, the brown rice Cd concentration in NIL-A3-P5 (∼0.11 mg/kg) was 79.3% lower than that in WSSM (∼0.53 mg/kg) in 2022, whereas that in L1S/NIL-A3-P5 (∼0.13 mg/kg) was 69.3% lower than that in L1S/WSSM (∼0.43 mg/kg) in 2023 (Figure 6N). Furthermore, compared with the WT, plants harboring *OsNRAMP5*^*LAA*^ consistently exhibited lower brown rice Cd concentrations (below 0.2 mg/kg, the maximum limit in China and the European Union) as well as lower Cd and Mn concentrations in straw. Brown rice Mn concentrations were lower or not significantly different among genotypes in Fields C and D (Figures 6K, 6L, 6O, and 6P; Supplemental Figures 13D and 13E). Moreover, yield per plant did not differ significantly among these NILs and WSSM, nor between L1S/WSSM and L1S/NIL-A3-P5 (Figure 6M; Supplemental Figure 13F) in Fields C and D. Because Field A was contaminated with Cd (soil total Cd, 1.2 mg/kg), we also measured Cd accumulation in plants grown at this site. Cd concentrations in straw and brown rice were 78.1% and 75.6% lower in NIL-A3-P5 than in WSSM, respectively (Supplemental Figure 13C). The brown rice Cd concentration in NIL-A3-P5 was ∼0.25 mg/kg, which is below the upper limit set by the Codex Alimentarius Commission (0.4 mg/kg) (Supplemental Figure 13C). These results suggest that *OsNRAMP5*^*LAA*^ is an elite haplotype for the genetic improvement of low-Cd rice.

## Discussion

Cd uptake by roots and transport to grains are mediated by transporters of mineral elements with similar physicochemical properties, such as Mn, Fe, and Zn (Zhao et al., 2022). Consequently, reducing Cd accumulation is often accompanied by a decrease in essential mineral elements. Substantially reducing grain Cd concentrations while maintaining appropriate mineral element levels remains a major bottleneck in low-Cd rice breeding (Yan et al., 2025). In rice, Cd uptake is mediated primarily by the Mn transporter OsNRAMP5, which contributes most to grain Cd accumulation identified to date. Moreover, OsNRAMP5 also mediates Fe uptake, but its contribution to Fe uptake is dispensable in rice (Ishikawa et al., 2012; Sasaki et al., 2012). Knockout of *OsNRAMP5* can minimize grain Cd concentrations; however, it renders rice sensitive to low-Mn and heat stress due to Mn deficiency rather than Fe deficiency (Sasaki et al., 2012; Yang et al., 2014; Dong et al., 2021). Here, we identified LAA, a tropical *japonica* rice cultivar with low-Cd grains and moderate Mn accumulation, and demonstrated that the genetically linked *OsNRAMP5*^*LAA*^ and *OsHMA3*^*LAA*^ alleles on the short arm of chromosome 7 are responsible for its low Cd accumulation (Figures 2A–2C). Notably, *OsNRAMP5*^*LAA*^, a novel allele of *OsNRAMP5*, reduced Cd and Mn uptake (Figures 3E–3H), leading to Cd and Mn accumulation levels intermediate between those of the WT and *osnramp5* (Figure 3A–3D), without affecting root-to-shoot Mn translocation (Supplemental Figures 4G and 5E). NIL-*OsHMA3*^*LAA*^ exibited a reduced root-to-shoot Cd translocation ratio as well as lower shoot/straw and grain Cd concentrations (Figures 3A and 3B; Supplemental Figure 5D; Figures 6J and 6K), indicating that *OsHMA3*^*LAA*^ is a strong allele. The elevated expression level of *OsHMA3*^*LAA*^, driven by promoter variations reported previously, contributes to its enhanced function (Figure 2D; Supplemental Figure 2C) (Liu et al., 2020). Moreover, its encoded protein carries multiple amino acid variations consistent with earlier findings (Liu et al., 2020). The effects of these amino acid substitutions on Cd transport activity warrant further validation. In hydroponic assays with 0.5 μM Cd, the reduction in shoot Cd concentration conferred by *OsNRAMP5*^*LAA*^ (56.3%) was 1.7 times that caused by *OsHMA3*^*LAA*^ (32.3%).

Mn is both an essential micronutrient for plants and a potent stress factor when accumulated excessively (Shao et al., 2017). Because of the high expression level and strong Mn uptake activity of OsNRAMP5, rice is a typical crop with high Mn accumulation (Sui et al., 2018). Additionally, rice is highly tolerant to Mn. Rice leaf Mn concentrations often exceed 1000 mg/kg DW—10–20 times the normal growth threshold—without causing symptoms of Mn toxicity. By contrast, maize growth is inhibited when leaf Mn concentrations reach 200 mg/kg (Shao et al., 2017). Under identical hydroponic conditions, the shoot Mn concentration of rice is approximately three times that of maize and twice that of wheat (Sui et al., 2018). Therefore, moderately reducing OsNRAMP5 function in rice would be expected to lower Cd concentrations without disrupting Mn homeostasis. Under 0.1–1 μM Mn, the *OsNRAMP5*^*Q337K*^ mutant displayed low-Mn tolerance comparable to that of the WT and superior to that of *osnramp5* (Kuramata et al., 2022). However, under 0.05 μM Mn, the *OsNRAMP5*^*Q337K*^ mutant showed reduced growth due to an approximately 50% decrease in leaf Mn concentration compared with the WT (slightly above 20 mg/kg) (Kuramata et al., 2022). In the present study, no significant differences were observed in plant height, root length, or shoot Mn concentration (slightly below 20 mg/kg) between NIL-*OsNRAMP5*^*LAA*^ and WT plants under 0.05 μM Mn (Supplemental Figures 6A–6C). These results imply that rice plants carrying *OsNRAMP5*^*LAA*^ tolerate lower Mn concentrations in the growth medium compared with those carrying *OsNRAMP5^Q337K^*. Under combined low-Mn and heat stress at the seedling stage, NIL-*OsNRAMP5*^*LAA*^ exhibited Mn-SOD activity comparable to that of the WT, and shoot Mn concentrations in NIL-*OsNRAMP5*^*LAA*^ and WT plants were within the normal range required for plant growth and development (50–100 mg/kg DW) (Figures 3O and 3P). Consequently, the stress tolerance of NIL-*OsNRAMP5*^*LAA*^ was similar to that of WSSM and significantly greater than that of *osnramp5* (Figures 3I–3N).

Deletion of certain regulatory sequences in the *OsNRAMP5* promoter impaired its translational efficiency, reducing grain Cd concentrations without negatively affecting Mn accumulation or grain yield (Luo et al., 2023). Nevertheless, grain Cd concentrations were reduced by at most 50%, and the underlying mechanism remained unclear. Intriguingly, tandem duplication of *OsNRAMP5* reduced grain Cd concentrations by 64% (Yu et al., 2022). However, the tolerance of rice varieties carrying this allele to high-Mn acidic soils warrants careful evaluation, given the markedly elevated Mn concentrations in their shoots and roots. Similarly, *OsNRAMP5* overexpression driven by the rice *Actin1* promoter or the maize *Ubiquitin* promoter enhanced Cd uptake in roots but reduced root-to-shoot Cd translocation, decreasing grain Cd concentrations by 49%–94% while also reducing yield by 22.9%–32.6% (Chang et al., 2020b). Recently, yeast-based assays have been used to identify specific amino acid substitutions in OsNRAMP5 that may confer differential uptake efficiencies for Cd and Mn. However, because OsNRAMP5 does not localize to the PM in yeast cells (Supplemental Figure 7C), the effects of these variants must be validated in rice plants (Qu and Nakanishi, 2023; Inoue et al., 2024). In our Cd-contaminated field trials, introgression of *OsNRAMP5*^*LAA*^ reduced grain Cd concentrations by 64.4%. Furthermore, pyramiding *OsHMA3*^*LAA*^ and *OsNRAMP5*^*LAA*^ decreased grain Cd concentrations by 71.0%–79.3% (Figures 6J and 6N; Supplemental Figure 13C), consistently maintaining Cd levels below the maximum limits set by the Codex Alimentarius Commission (0.4 mg/kg) or by China and the European Union (0.2 mg/kg). Knockout of the phosphate transporter gene *OsPT1* or its transcription factor *OsbHLH35* indirectly reduced the transcription of several Fe/Cd, Mn/Cd, and Zn/Cd transporter genes by altering ion homeostasis, leading to a comparable reduction in grain Cd concentrations. However, it remains unclear whether the reduced micronutrient levels in these mutants are sufficient to support stress tolerance in rice (Peng et al., 2025). Notably, combining *OsNRAMP5*^*LAA*^ and *OsHMA3*^*LAA*^ with *osnramp5* in hybrid rice resulted in a substantial reduction in grain Cd concentrations without compromising grain yield (Figures 6J and 6M), providing an effective alternative approach for breeding low-Cd rice. When exposed to high temperatures or combined low soil Mn and heat stress during the booting and heading stages, *osnramp5* mutants showed substantial yield losses, whereas NILs harboring *OsNRAMP5*^*LAA*^ and *OsHMA3*^*LAA*^ maintained grain yields comparable to those of the WT (Figure 6D). In low-Mn paddy fields, straw Mn concentrations in NILs carrying *OsNRAMP5*^*LAA*^ and *OsHMA3*^*LAA*^ were approximately 148.6 mg/kg, exceeding the requirement for normal growth and development (50–100 mg/kg DW) (Mitani-Ueno et al., 2018), whereas straw Mn concentrations in *osnramp5* lines ranged from 64.1 to 71.9 mg/kg, within the lower end of this range (Figure 6I). These results suggest that rice plants require higher leaf Mn levels to withstand heat and low-Mn stress during reproductive growth. These results further indicate that even under combined low-Mn and heat stress during reproductive development, Mn uptake mediated by OsNRAMP5^LAA^ is sufficient to maintain leaf Mn concentrations, thereby preserving yield and stress resilience. Notably, *OsNRAMP5*^*LAA*^ is a rare natural allele, accounting for only ∼0.27% of the 3K-RG population (Figure 2J), highlighting its strong potential for developing low-Cd and broadly adaptable rice cultivars. The high conservation of S^313^ within the NRAMP family further suggests a potentially universal role for this residue in ER exit, providing insights for developing low-Cd crop cultivars through targeted mutagenesis of corresponding residues in orthologous proteins.

**Figure 7:**
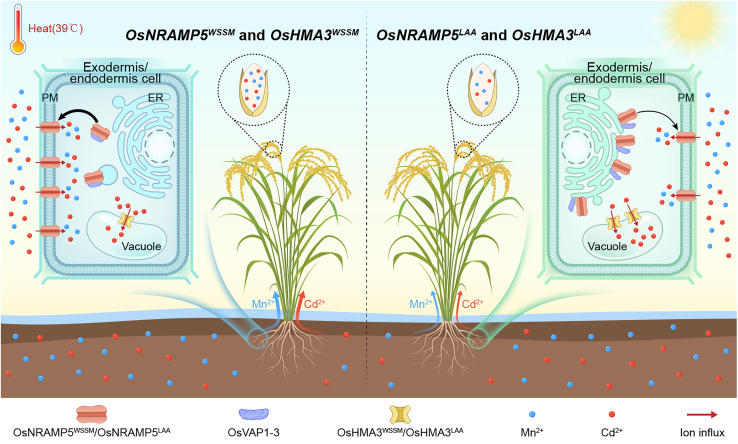
Working model illustrating the roles of OsNRAMP5^LAA^ and OsHMA3^LAA^ in reducing grain Cd concentrations. OsNRAMP5 is an influx transporter localized to the plasma membrane (PM) of both exodermal and endodermal cells in roots and is primarily responsible for Cd and Mn uptake. The VAMP-associated protein OsVAP1-3 interacts with OsNRAMP5 in the endoplasmic reticulum (ER) and facilitates its export from the ER (left). In the tropical *japonica* cultivar LAA, the interaction between OsNRAMP5^LAA^ and OsVAP1-3 is weakened, resulting in partial retention of OsNRAMP5^LAA^ in the ER and consequently limiting Cd and Mn uptake (right). Notably, Mn uptake mediated by OsNRAMP5^LAA^ remains sufficient to maintain adequate leaf Mn levels to withstand heat and low-Mn stress. In addition, OsHMA3^LAA^ sequesters more Cd into the vacuoles of root cells than OsHMA3^WSSM^. The combination of reduced Cd uptake mediated *OsNRAMP5*^*LAA*^ and enhanced vacuolar Cd sequestration by *OsHMA3*^*LAA*^, substantially lowers grain Cd concentrations without compromising stress tolerance in rice.

*OsNRAMP5* transcription is not significantly affected by variations in Cd and Mn concentrations in the growth medium (Sasaki et al., 2012; Yang et al., 2014; Yu et al., 2022), whereas its post-transcriptional regulation remains largely unclear. AtNRAMP1, a Mn and Cd transporter in *Arabidopsis thaliana*, shuttles between the PM and internal vesicular compartments in response to fluctuations in environmental Mn, a process regulated by the phosphorylation status of S at position 20 on its N-terminus (Castaings et al., 2021). Cd stress induces accumulation of the receptor-like kinase AtWAKL4, leading to phosphorylation of AtNRAMP1 and promoting its endocytosis from the PM and degradation in vacuoles, thereby reducing Cd uptake and accumulation (Yuan et al., 2024). However, the general landscape of intracellular trafficking from the ER to the final destinations of plant NRAMP family members remains largely unknown. Here, we found that the OsNRAMP5^LAA^ protein carries a single amino acid substitution, S313F. This variation weakens Cd and Mn uptake while maintaining the root-to-shoot Mn translocation function of OsNRAMP5 (Supplemental Figures 4D–4G). Correct subcellular localization of transporters is critical for their physiological function, particularly in mediating substrate transport across membranes. We identified a novel VAMP-associated protein, OsVAP1-3, which binds to OsNRAMP5 in the ER and facilitates its export from the ER (Figures 5A–5F). Notably, the S313F substitution in OsNRAMP5 weakens its interaction with OsVAP1-3 in the ER and impairs ER exit, resulting in partial ER retention and reduced trafficking of OsNRAMP5 to the PM for mediating Cd and Mn uptake (Figures 5A–5F and 4F–4K; Supplemental Figure 11C). Consequently, Cd and Mn uptake in rice is reduced, yet Mn uptake mediated by OsNRAMP5^S313F^ remains sufficient for rice to withstand high temperatures and Mn deficiency (Figure 7). Our results therefore demonstrate that ER export of OsNRAMP5, aided by OsVAP1-3, is a critical determinant of Cd and Mn uptake in rice. The S^313^ residue of OsNRAMP5 is not only involved in binding to multiple OsVAP proteins but is also crucial for efficient ER exit of OsNRAMP5 (Figures 5A–5F and 4F–4K; Supplemental Figure 12B). Membrane proteins often contain short ER export signal motifs that are cytoplasmically exposed and required for recruitment into coat protein complex II (COPII)-coated vesicles that mediate ER export (Nufer et al., 2002; Zhu et al., 2023). These ER export motifs are generally classified into acidic and hydrophobic motifs (Zhu et al., 2023). The finding that S313F inhibits ER export of OsNRAMP5 suggests the presence of an important ER export signal motif in the vicinity of the S^313^ residue. Moreover, the Cd and Mn uptake function of OsNRAMP5 relies on its polar localization to the distal sides of exodermis and endodermis cells (Sasaki et al., 2012). It was recently reported that OsNRAMP5 mediates xylem unloading of Mn from the leaf sheath, a process crucial for leaf sheath growth (Huang et al., 2023). Whether the S313F substitution affects the polar localization of OsNRAMP5 in root cells or Mn unloading from leaf sheath xylem warrants further investigation.

Ongoing global warming increases the likelihood of heat stress and water shortages in agricultural systems. Additionally, as soils become drier, Mn bioavailability declines. These scenarios underscore the need for rice cultivars with low-Cd grains that also exhibit strong tolerance to low-Mn and heat stress. Here, we demonstrate that pyramiding the genetically linked *OsNRAMP5*^*LAA*^ and *OsHMA3*^*LAA*^ alleles substantially reduces grain Cd accumulation while maintaining sufficient Mn uptake to confer resistance to low-Mn and heat stress. Our findings provide valuable genetic resources and a novel approach for developing rice cultivars that balance low grain Cd accumulation with stress tolerance.

## Methods

### Plant materials and growth conditions

LAA is a tropical *japonica* rice cultivar from Indonesia. The *japonica* rice cultivar Nip was used as a low-Cd control, whereas two commercial *indica* rice cultivars, WSSM and HZ, were used as high-Cd controls. The 20 representative rice cultivars used to measure grain Cd and Mn concentrations are listed in Supplemental Table 1. *OsNRAMP5* and *OsVAP1-3* knockout plants were generated using CRISPR–Cas9 technology and the corresponding single-guide RNA sequences are listed in Supplemental Table 3. OsNRAMP5^S313F^ and OsNRAMP5^S313A^ plants generated by prime editing were obtained following a previously reported method (Li et al., 2022b). The prime editing guide RNA (pegRNA) sequences are listed in Supplemental Table 3.

For hydroponic experiments, rice seeds were soaked in water for 2 days and germinated for 16 h at 37°C. Germinated seeds were transferred to 96-well PCR plates with the bottoms removed, and the plates were floated on half-strength Yoshida nutrient solution (Yoshida et al., 1976). The nutrient solution was renewed every 2 days. Plants were grown in a growth chamber under a 16-h light (28°C) and 8-h dark (25°C) photoperiod. At the end of hydroponic culture with Cd or Mn treatments, seedlings were washed three times with 0.5 mM CaCl_2_ followed by deionized water and then separated into shoots and roots for determination of metal concentrations.

Field trials were conducted in experimental paddy fields in Hunan Province, China. In the paddy fields located in Changsha (Field D), Zhuzhou (Field C), and Hengyang, total Cd concentrations in soil were 0.9, 0.5, and 1.1 mg/kg; total Mn concentrations in soil were 385.2, 433.7, and 329.6 mg/kg; and soil pH values were 5.8, 5.6, and 5.9, respectively. To assess rice tolerance to heat and low-Mn stress, plants were sown in experimental paddy Fields A and B in Changsha on May 31, 2022. In Fields A and B, soil total Mn concentrations were 312 and 102 mg/kg, soil total Cd concentrations were 1.2 and 0.1 mg/kg, and soil pH values were 6.6 and 6.5, respectively. At maturity, aboveground plant parts were harvested by cutting 10 cm above the soil surface and separated into straw and grains. Grains were dehusked to obtain brown rice. Straw samples were washed three times with tap water followed by deionized water and used for determination of metal concentrations.

### Map-based cloning

Primary gene mapping was performed using an F_2_ population derived from a cross between WSSM and LAA. A total of 456 F_2_ plants and 12 parental plants exposed to 0.5 μM Cd for 12 days were used to determine shoot Cd concentrations. Two pools of F_2_ plants with extreme shoot Cd concentrations (30 individuals each with low and high shoot Cd concentrations, respectively) were established. Genomic DNA was extracted from the roots of plants in the two pools and their parental lines for BSA sequencing. The differences in SNP and indel indices between the two pools were calculated. A QTL region was identified by comparing and subtracting the SNP and indel index values of the low-Cd pool from those of the high-Cd pool. For fine mapping of causal genes, six KASP markers (M297–M303) within this region were developed (Supplemental Table 2). An enlarged F_2_ population containing 1265 plants was screened for recombinants using these KASP markers. Plants with recombination between *OsNRAMP5* and *OsHMA3* or between *OsNRAMP5* and *OsNRAMP1* were isolated from the F_2_ population, and homozygous recombinant plants were generated by self-pollination to produce F_2:3_ progeny. Shoot Cd concentrations of these progeny were then determined.

### Generation of near-isogenic lines (NILs)

WSSM and LAA were used as the recipient and donor parents, respectively. Through continuous backcrossing and self-pollination, combined with the use of KASP markers M297 and M302 (Figure 2A) and multiple nucleotide polymorphism DNA markers evenly distributed across the 12 rice chromosomes (Fang et al., 2021), NIL-*OsNRAMP5*^*LAA*^-*OsHMA3*^*LAA*^ in the WSSM background was selected in the BC_4_F_2_ generation. To obtain single-gene NILs, NIL-*OsNRAMP5*^*LAA*^-*OsHMA3*^*LAA*^ was further backcrossed with WSSM, followed by self-pollination. Using KASP markers M297, M299, M302, and M303 flanking *OsHMA3* or *OsNRAMP5* (Figure 2A), heterozygous plants with recombination between *OsHMA3* and *OsNRAMP5* were identified from the BC_1_F_1_ population. Subsequently, homozygous NIL-*OsNRAMP5*^*LAA*^ and NIL-*OsHMA3*^*LAA*^ lines were obtained in the BC_1_F_2_ generation using KASP markers. Primers used for all KASP markers are listed in Supplemental Table 2.

### Analysis of natural variations in *OsNRAMP5*

SNP data for *OsNRAMP5* CDSs in 3024 rice cultivars were downloaded from the Rice SNP-seek Database (https://snpseekv3.duckdns.org/). Accessions with heterozygous variants or missing data due to insufficient sequencing coverage were excluded. Consequently, 2605 accessions were analyzed. Haplotype networks were constructed using Network software (v5.0).

### Analyses of metal concentrations and metal translocation ratio

Samples were dried at 70°C for 3 days and ground into powder. Powdered samples were used to determine metal concentrations via inductively coupled plasma mass spectrometry (Agilent 7700 series, USA) according to a previously reported method (Tang et al., 2022). Cd and Mn root-to-shoot translocation ratios were calculated as the percentage of total metal accumulated in shoots relative to the total metal accumulated in the whole plant, as described previously (Tang et al., 2022).

### Short-term Cd and Mn uptake assays

Short-term Cd uptake assays were conducted using 4-week-old seedlings according to a previously established method (Tang et al., 2022). For short-term Mn uptake assays, 3-week-old seedlings were transferred to a nutrient solution containing 0.05 μM Mn for 1 week. The seedlings were then exposed to a pretreatment solution containing 0.5 mM CaCl_2_ and 2 mM MES for 12 h, and then transferred to an uptake solution containing 0.5 mM CaCl_2_, 2 mM MES, and various concentrations of Mn (0, 0.2, 0.5, 5, 10, 50, and 100 μM) at 26°C and 4°C. After 20 min, plant roots were immersed in an ice-cold pretreatment solution for 5 min and rinsed three times with deionized water. Roots were then dried at 70°C for 3 days and used for Mn determination as described above.

### Thermotolerance assays

Uniformly germinated seeds were cultured under 0.2 or 9 μM Mn for 14 days using the hydroponic method described above. Seedlings were then transferred to a growth chamber at 45°C for 3 days and returned to a growth chamber at 28°C for 7 days. Cultivation at 28°C with the corresponding Mn supply for the same period served as the control.

Shoots of seedlings subjected to heat stress at 45°C for 6 h and those under control conditions were sampled, and Mn-SOD activity was measured using the SOD Isozyme Activity Detection Kit (Solarbio, China). After3 days of heat (45°C) or control treatment, shoots were harvested to measure MDA and Mn contents using the MDA Content Assay Kit (Solarbio) and the method described above, respectively. The third leaf blade of seedlings was used to visualize O_2_^−^ levels via NBT staining using the Plant Tissue Reactive Oxygen Species Assay Kit (Coolaber, China). After 7 days of recovery, representative plants were photographed, and seedling survival rates were calculated (24 seedlings per replicate).

### Gene expression analysis

Total RNA was extracted using the Eastep Super Total RNA Extraction Kit (Promega, USA) according to the manufacturer’s instructions. First-strand cDNA was synthesized from total RNA using PrimeScript RT Master Mix (Takara). Quantitative real-time PCR (real-time qPCR) was performed using TB Green Premix Ex Taq II Mix (Takara) on a Light Cycler 480 thermocycler (Roche). *OsActin* (Os03g0718100) was used as the endogenous reference gene, and relative gene expression was calculated using the 2^−ΔΔCt^ method. All experiments were conducted with three biological and three technical replicates. Primers used for real-time qPCR are listed in Supplemental Table 3.

### Protein structure and sequence conservation analyses

The amino acid sequence of OsNRAMP5 was analyzed using Phyre2 (Kelley et al., 2015), and the predicted secondary structure and transmembrane helix positions were visualized using TMRPres2D (Spyropoulos et al., 2004). To analyze the conservation of the S313 residue and its flanking sequence, homologous protein sequences of OsNRAMP5 were identified via a BLAST search of the UniProt database (https://www.uniprot.org/), using the amino acid sequence spanning positions 292–317 of OsNRAMP5 as the query. These sequences were then analyzed using MEGA software (v7.0), and the degree of conservation of each amino acid was determined using pLOGO (https://plogo.uconn.edu/).

### Subcellular localization

The CDSs of *OsNRAMP5*, *OsNRAMP5^S313F,^* and *OsVAP1-3* (lacking stop codons) were amplified by PCR from root cDNA of WSSM or LAA and cloned into the pYBA1132-*EGFP* vector containing the 35S promoter for subcellular localization assays in rice protoplasts and tobacco leaves, respectively. Primers are listed in Supplemental Table 3. HEDL-mCherry and AtPIP2A-mCherry were used as ER and PM markers, respectively. For rice protoplast transformation, the desired plasmid combinations (Figure 4F) were co-transformed into protoplasts isolated from etiolated rice seedlings using the polyethylene glycol-mediated method and incubated at 28°C in the dark for 18 h. For transient expression in tobacco leaves, plasmid combinations (Figure 4G) were co-transformed into *N. benthamiana* leaves using *Agrobacterium*-mediated transformation. Transformed plants were cultured for 2 days. For subcellular localization analysis of OsNRAMP5 and OsNRAMP5^S313F^ in rice root cells, binary vectors p*Ubi:OsNRAMP5-EGFP* and p*Ubi:OsNRAMP5*^*S313F*^*-EGFP* driven by the *Ubiquitin* promoter were constructed and transformed into the *osnramp5* cultivar LH3B using *Agrobacterium*-mediated transformation. After germination, T_2_ homozygous rice lines were grown in nutrient solution containing 9 or 0.2 μM Mn for 6 days and then treated with or without 0.25 μM Cd for 1 h. Roots were stained with 10 μM FM4-64, a lipophilic styryl dye, in the dark for 10 min to visualize the PM (red fluorescence). Fluorescence signals were detected using a confocal laser scanning microscope (LSM880, Zeiss, Germany). The mean GFP fluorescence intensity in the PM and ER of the same root cell in OsNRAMP5^S313F^-EGFP plants was analyzed using ImageJ software, and the ratio of mean OsNRAMP5^S313F^ fluorescence intensity in the ER to that in the PM was calculated.

### Transporter activity assay in yeast

The CDSs of *OsNRAMP5* and *OsNRAMP5*^*S313F*^ were cloned into pYES2 vectors. pYES2 contains the *GAL1* promoter, which induces gene expression in the presence of galactose but represses it in the presence of glucose. These plasmids were transformed into *Δycf1* strains (Cd-sensitive yeast mutant) and *Δsmf1* strains (yeast mutant defective in Mn uptake) using the Frozen Yeast Transformation Kit (Coolaber). Transformed yeast strains were cultured in uracil-omitted synthetic defined (SD–Ura) liquid medium containing 2% glucose to the logarithmic phase, harvested by centrifugation, and washed three times with water. The OD_600_ values of yeast suspensions were adjusted to 0.5 with sterile water and then serially diluted 10-fold three times. Subsequently, 6 μl of yeast suspension from each dilution was spotted onto SD-Ura solid medium containing 2% glucose or 2% galactose supplemented with 0, 15, or 20 μM Cd (for the *Δycf1* strain) or 0, 2, or 3 mM EGTA (for the *Δsmf1* strain). Plates were incubated at 30°C for 3 days. Growth phenotypes of yeast cells were then observed and photographed.

For subcellular localization analysis of OsNRAMP5 and OsNRAMP5^S313F^ in yeast, the CDSs of *OsNRAMP5*-*EGFP* and *OsNRAMP5*^*S313F*^-*EGFP* were amplified by PCR and cloned into pYES2 vectors. The resulting plasmids were transformed into yeast cells. Transformed yeast strains were pre-cultured overnight in SD–Ura liquid medium containing 2% glucose, washed with water, and then grown in SD–Ura liquid medium containing 2% galactose for 9 h. Subsequently, GFP fluorescence was observed using a confocal microscope. To localize OsNRAMP5 to the PM of yeast cells, the nucleotide sequence encoding the C-terminal domain of yeast IST2 (aa 589–946) was amplified by PCR and inserted into the pYES2 vector to generate the pYES2*–IST2*^*C*^ vector. The CDSs of *OsNRAMP5*, *OsNRAMP5*^*S313F*^, *OsNRAMP5-EGFP,* and *OsNRAMP5*^*S313F*^*-EGFP* (lacking stop codons) were then amplified by PCR from the corresponding pYES2 vectors and cloned into pYES2-*IST2*^*C*^ vectors, where they were fused in-frame with the IST2^C^ domain. Primers are listed in Supplemental Table 3. Yeast spot assays and subcellular localization observations were performed as described above. Growth curves of yeast cells harboring the indicated plasmids or an empty vector were monitored in liquid SD–Ura medium with or without 30 μM Cd or 12.5 mM EGTA in the presence of 2% galactose. Initial OD_600_ values of these yeast transformants were identical, ranging from 0.05 to 0.1. OD_600_ values were measured every few hours from the early growth phase to the plateau phase.

### Y2H assay

The CDS of the loop linking the seventh and eighth transmembrane domains of OsNRAMP5 was cloned into the pBT-N vector to generate the bait vector BD-Loop. Total RNA was extracted from roots of WSSM seedlings grown in standard hydroponic solution using the RNeasy Plant Mini Kit (Qiagen, Germany). Construction of the root cDNA library and screening for proteins interacting with BD-Loop were performed using the split-ubiquitin membrane-based Y2H system. CDSs of selected *OsVAP family* members were cloned into the pPR3-N vector to generate the corresponding prey vectors. CDSs of OsNRAMP5, OsNRAMP5^S313F,^ and Loop^S313F^ were cloned separately into the pBT-N vector to generate the corresponding bait vectors. Different combinations of bait and prey vectors were co-transformed into yeast strain NMY51, and positively transformed colonies were selected after incubation on synthetic medium lacking Leu and Trp at 30°C for 3 days. Transformed yeast colonies were serially diluted (initial OD_600_ of 1.0) and cultured on synthetic medium lacking Leu, Trp, His, and Ade with or without X-gal at 30°C for 4 days. Protein–protein interactions were assessed by observing growth phenotypes. β-Galactosidase activity was measured using a Yeast β-Galactosidase Assay Kit (Thermo Fisher Scientific, USA). The experiment was repeated at least three times with similar results.

### BiFC assay

The CDS of *OsNRAMP5* or *OsNRAMP5*^*S313F*^ was cloned into the *proMAS:cYFP* vector (containing the C-terminus of YFP) to generate *OsNRAMP5-cYFP* or *OsNRAMP5*^*S313F*^*-cYFP*, respectively. The CDS of OsVAP1-3 was cloned into the *proMAS:nYFP* vector (containing the N-terminus of YFP ) to generate *nYFP-OsVAP1-3*. Various combinations of BiFC vectors (Figure 5E) and HDEL-mCherry vectors were co-transformed into rice protoplasts using the polyethylene glycol-mediated method. After overnight incubation in the dark, YFP fluorescence was observed and photographed using a confocal laser scanning microscope (LSM880, Zeiss, Germany). Combinations of OsNRAMP5-cYFP and nYFP, OsNRAMP5^S313F^-cYFP and nYFP, and cYFP and nYFP-OsVAP1-3 were used as negative controls.

### Split-LUC assay

CDSs of Loop, Loop^S313F^, and GUS were cloned into the pCAMBIA-nLUC vector (containing the N-terminus of LUC). CDSs of *OsVAP1-3* and *GUS* were cloned into the pCAMBIA-cLUC vector (containing the C-terminus of LUC). These plasmids were independently transformed into *Agrobacterium tumefaciens* strain GV3101. Transformed *Agrobacterium* cells were resuspended in infiltration buffer (10 mM MES, 10 mM MgCl_2_, and 150 μM acetosyringone) and adjusted to an OD_600_ of 1.5. *Agrobacterium* strains harboring the desired plasmids (Figure 5B) were mixed in equal proportions, and fully expanded *N. benthamiana* leaves were infiltrated with the mixture using needleless syringes. Infiltrated plants were grown for 2 days. Transformed leaves were then sprayed with 1 mM luciferin solution and incubated in the dark for 5 min. LUC signals were detected using an *in vivo* imaging system (Vilber Newton 7.0, France). For quantitative analysis, samples collected from transformed leaves were analyzed using a microplate luminometer. To avoid transformation efficiency bias, at least 10 individual leaves from different plants were used for each combination. All experiments were repeated three times with similar results.

### Co-immunoprecipitation assay

Vector combinations of *pro35S:OsNRAMP5-GFP* and *pro35S:OsVAP1-3-3FLAG*, or *pro35S:GFP* and *pro35S:OsVAP1-3-3FLAG*, were co-transformed into rice protoplasts using the polyethylene glycol-mediated method. After 12 h of incubation, ER proteins were extracted from rice protoplasts using the Minute Plant ER Enrichment Kit (Invent Biotechnologies, USA) and immunoprecipitated with anti-GFP immunomagnetic beads according to a previously described method (Liu et al., 2024). Samples were subjected to immunoblot analysis using anti-GFP (Abclonal, USA) and anti-FLAG (Abclonal, USA) antibodies. *pro35S*:GFP coexpressed with OsVAP1-3-3FLAG was used as a negative control. Band intensities were analyzed using Image J software.

### Non-invasive micro-test technology (NMT)

Ten-day-old hydroponically grown seedlings were soaked in test solution (50 μM CaCl_2_ and 500 μM CdCl_2_ [pH 5.5]) for 15 min. The root maturation zone, approximately 900 μm from the root apex, was used to determine net Cd^2+^ flux using the NMT system (NMT150-IM-XY; Xu Yue Technology, China). Data were recorded for 10 min. Each measurement was repeated three times, with three biological replicates per line.

### PSII photochemistry measurement

To determine the maximum quantum efficiency of PSII (Fv/Fm), rice seedlings subjected to different treatments were dark-adapted for 30 min. Photosynthetic parameters of the third leaf were measured and imaged using PAM fluorescence imaging (IMAGING-PAM M-Series, Walz).

### Data analysis

Student’s *t*-test (∗*P* < 0.05 and ∗∗*P* < 0.01) and one-way ANOVA (*P* < 0.05) were used for statistical analyses using DPS software (v.9.01). All data are presented as means ± SD. Graphs were generated using GraphPad Prism (v8).

### Accession numbers

Sequence data from this article are available at the Rice Genome Annotation Project (https://rice.uga.edu/index.shtml) under the following accession numbers: *OsNRAMP5* (LOC_Os07g15370), *OsHMA3* (LOC_Os07g12900), *OsNRAMP1* (LOC_Os07g15460), and *OsVAP1-3* (LOC_Os08g05890).

## Funding

This project was supported by the 10.13039/501100001809National Natural Science Foundation of China (32201727), the 10.13039/501100012166National Key Research and Development Program of China (2024YFD2301401), and the Agricultural Science and Technology Innovation Fund Project of Hunan Province (2025CX77).

## Acknowledgments

We thank Prof. Jianlong Xu (Institute of Crop Science, Chinese Academy of Agricultural Sciences) for providing partial cultivars from the 3K-RG panel. We are grateful to Academician Daoxin Xie (Tsinghua University) and Prof. Chuanqing Sun (China Agricultural University) for valuable discussions and constructive suggestions on the manuscript. We also thank Navogene Company (Beijing, China) for performing BSA sequencing. No conflict of interest is declared.

## Author contributions

B.Z., C.C., L.B., and L.T. designed the research. L.T., J.W., and Z.J. performed most of the experiments. X. Li and X.H. constructed the vectors and characterized plant phenotypes. X. Liu and P.W. generated the prime-editing plant lines. Q.L. provided the rice cultivars and conducted the haplotype analysis. Y.L., B.M., Y.S., Z.W., and Y.P. contributed to the field trials. L.T. wrote the manuscript. B.Z. and C.C. revised the manuscript.

## Supplemental information

Document S1. Supplemental Figures 1–13 and Supplemental Tables 1–3

Document S2. Article plus supplemental information
