# Supplementary material for: Pyramiding elite alleles of the genetically linked OsNRAMP5 and OsHMA3 confers low Cd accumulation in rice grains without compromising stress tolerance
Source: Plant Commun. 2025 Dec 30;7(4):101690. doi: 10.1016/j.xplc.2025.101690 (PMC13084080; doi:10.1016/j.xplc.2025.101690)
Supplement: Document S1. Supplemental Figures 1–13 and Supplemental Tables 1–3 [file mmc1.pdf]

Plant Communications, Volume 7

Supplemental information

Pyramiding elite alleles of the genetically linked *OsNRAMP5* and *OsHMA3* confers low Cd accumulation in rice grains without compromising stress tolerance

Li Tang, Jiao Wang, Zhongying Ji, Xingrong Li, Xiaoshuang Liu, Qiming Lv, Pengcheng Wei, Xianlan Hu, Yaokui Li, Bigang Mao, Ye Shao, Yan Peng, Zhongwei Wei, Lianyang Bai, Caiyan Chen, and Bingran Zhao

Supplemental figures

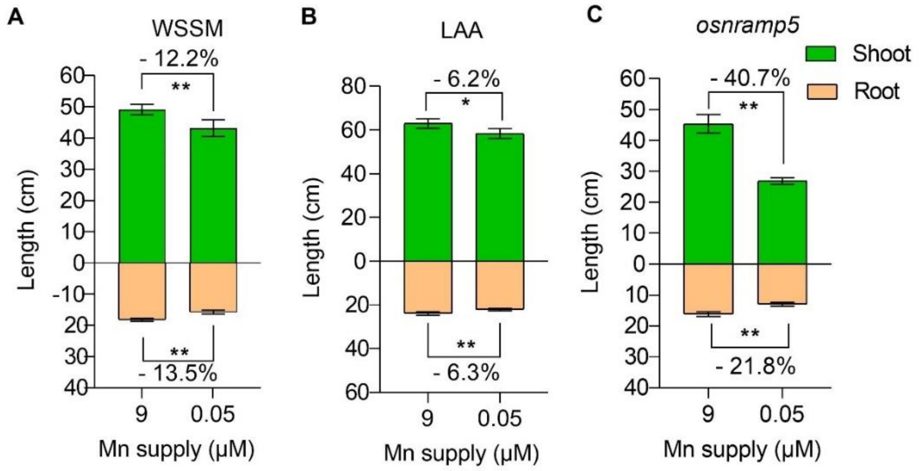

Supplemental Figure 1. Seedlings of LAA showed low-Mn stress tolerance.
(A-C) LAA, WSSM, and *osnramp5* in WSSM background were treated as described in the legend of Figure 1 G–N. The plants of WSSM (**A**)**,** LAA (**B**) and *osnramp5* (**C**) were sampled to measure plant height and root length. Values are presented as mean ± SD of five biological replicates. One or two asterisks indicate significant differences at $P < 0.05$ or $P < 0.01$, respectively, by Student's *t*-test.

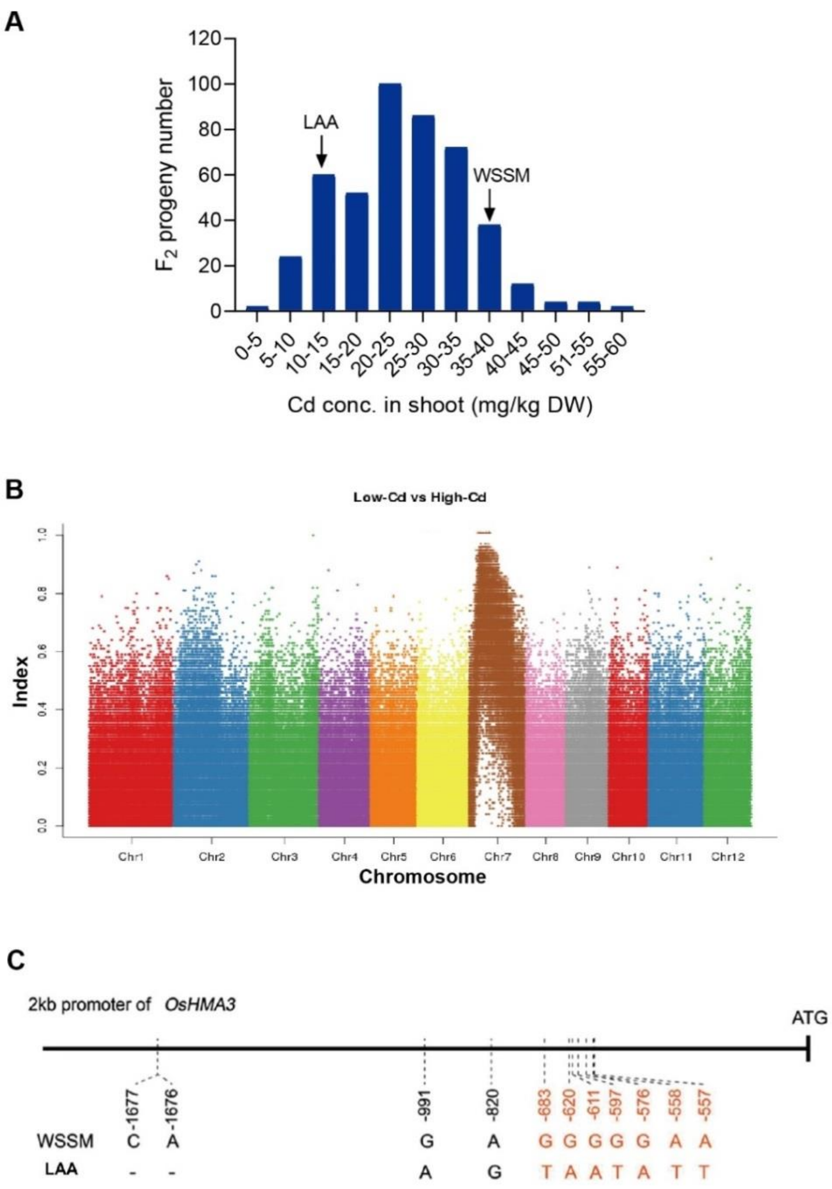

Supplemental Figure 2. QTL analysis of LAA, a low-Cd rice cultivar.

(A) Frequency distribution of shoot Cd concentrations of 456 F_2 seedlings derived from a cross between LAA and WSSM. Seedlings were grown in standard nutrient solution for 14 days and then treated with 0.5 μM Cd for additional 12 days. The shoot Cd concentrations of LAA and WSSM are indicated by black arrows. Conc., concentration; DW, dry weight.

(B) Distribution of InDel and SNP determined using bulked segregant analysis coupled with whole-genome sequencing.

(C) Sequence polymorphism in the 2 kb promoter of *OsHMA3* between WSSM and LAA.

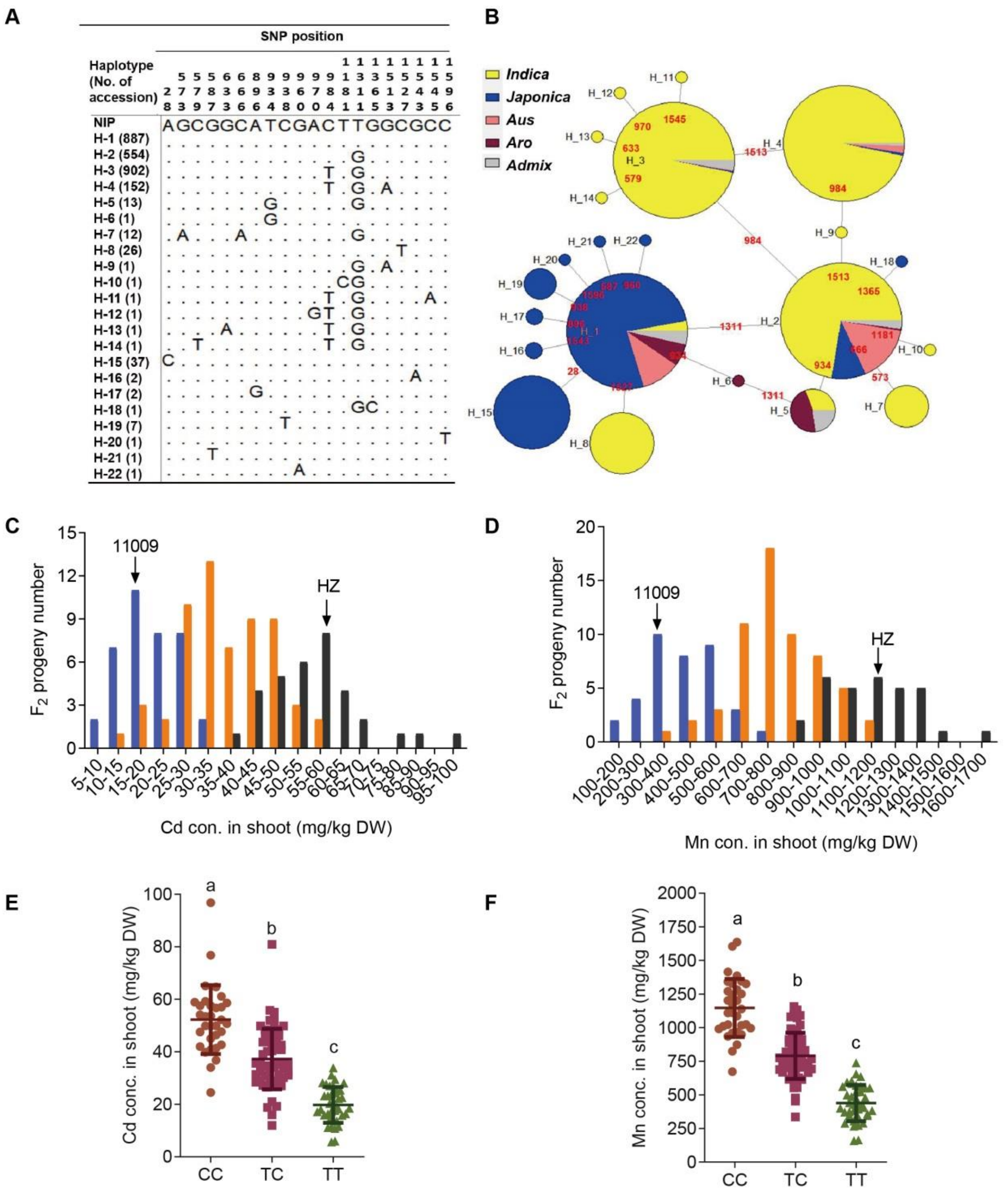

Supplemental Figure 3. Haplotype analysis of *OsNRAMP5* CDS and co-segregation analysis between the genotypes of 938th nucleotide in *OsNRAMP5* and the phenotypes of shoot Cd and Mn concentrations.

(A) Haplotype analysis of *OsNRAMP5* CDS from 2605 rice cultivars. Capital letters indicate variant nucleotides**.**

(B) Haplotype networks of different *OsNRAMP5* CDS. The circle size is proportional to the number of rice accessions harboring a given haplotype. Branch length represents the genetic distance between two haplotypes.

(C and D) Frequency distribution of shoot Cd **(C)** and Mn **(D)** concentrations in the F_2 population derived from a cross between the rice cultivars 11009 and HZ. The

seedlings were treated as described in the legend of Supplemental Figure 2A. The blue, orange, and black bars represent the frequencies of plants harboring homozygous TT, heterologous TC, and homozygous CC at position 938 of the *OsNRAMP5* CDS, respectively. The shoot Cd and Mn concentrations of 11009 and HZ are indicated by black arrows. Conc., concentration. DW, dry weight.

(E and F) Scatter plots of Cd **(E)** and Mn **(F)** concentrations in shoots of F_2 seedlings harboring different genotypes of 938th nucleotide in *OsNRAMP5* CDS. Different lowercase letters indicate significant differences by one-way ANOVA with Tukey's test ($P < 0.05$).

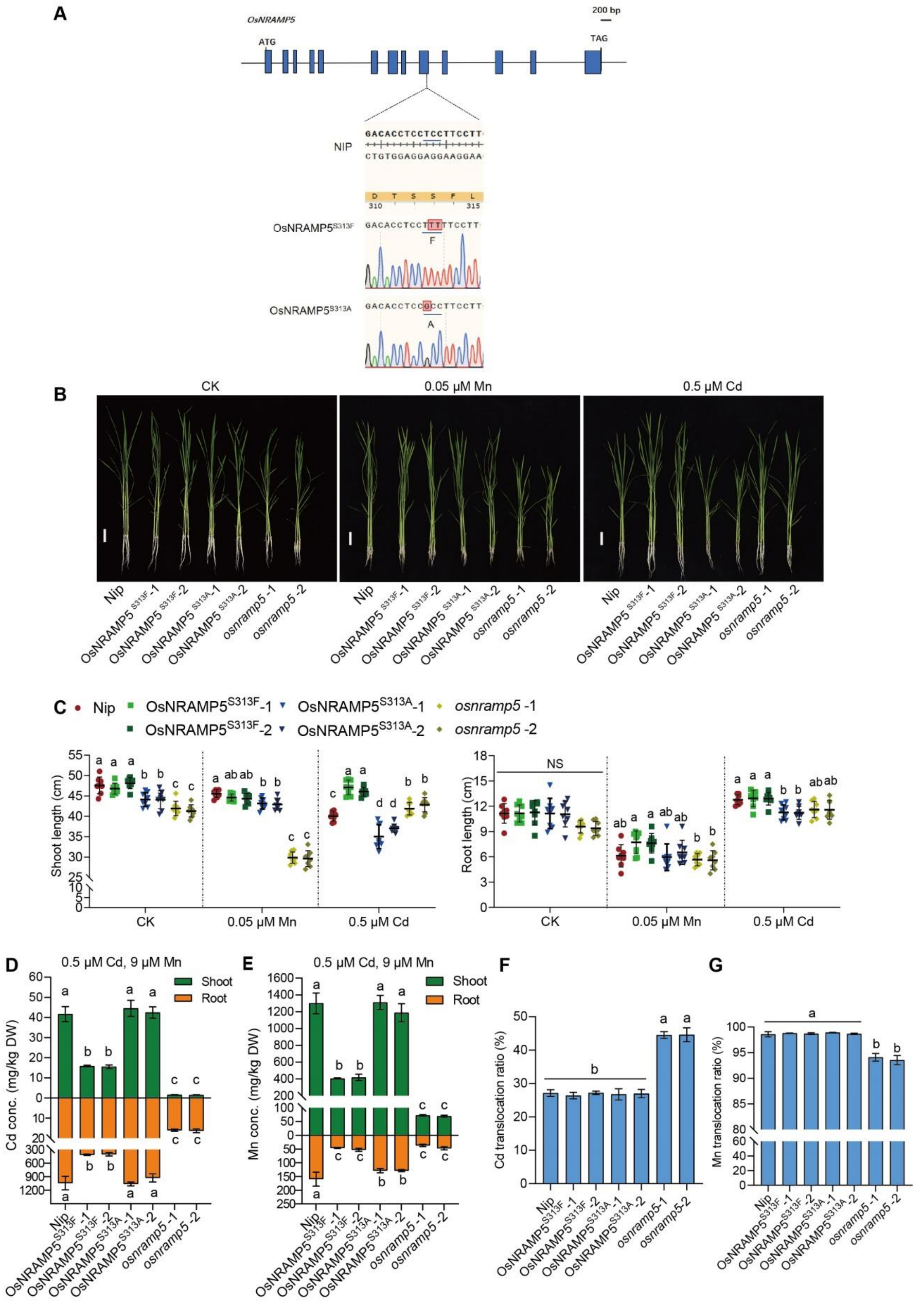

Supplemental Figure 4. The S313F substitution limits Cd and Mn uptake of OsNRAMP5.

(A) Identification of OsNRAMP5^{S313F} and OsNRAMP5^{S313A} plants in Nip background generated by prime editing.

(B–C) After germination, seeds of OsNRAMP5^{S313F}, OsNRAMP5^{S313A}, *osnramp5*,

and wild-type Nip were grown in the standard nutrient solution with 9 μM Mn for 28 days (CK), or grown in the standard nutrient solution with 9 μM Mn for 3 days and then transferred to the nutrient solution with 0.05 μM Mn for 25 days (0.05 μM Mn), or grown in the standard nutrient solution for 14 days and then exposed to 0.5 μM Cd for additional 14 days (0.5 μM Cd). These plants were photographed (**B**), and their shoot height and root length were measured **(C)**. Scale bar, 3 cm.

(D and E) Cd **(D)** and Mn **(E)** concentrations in shoots and roots in the presence of 0.5 μM Cd.

(F and G) The root-to-shoot translocation ratios of Cd **(F)** and Mn **(G)** were calculated.

Values are presented as means ± SD of four **(D–G)** to eight **(C)** biological replicates. Different lowercase letters indicate significant differences by one-way ANOVA with Tukey's test ($P < 0.05$). NS, not significant.

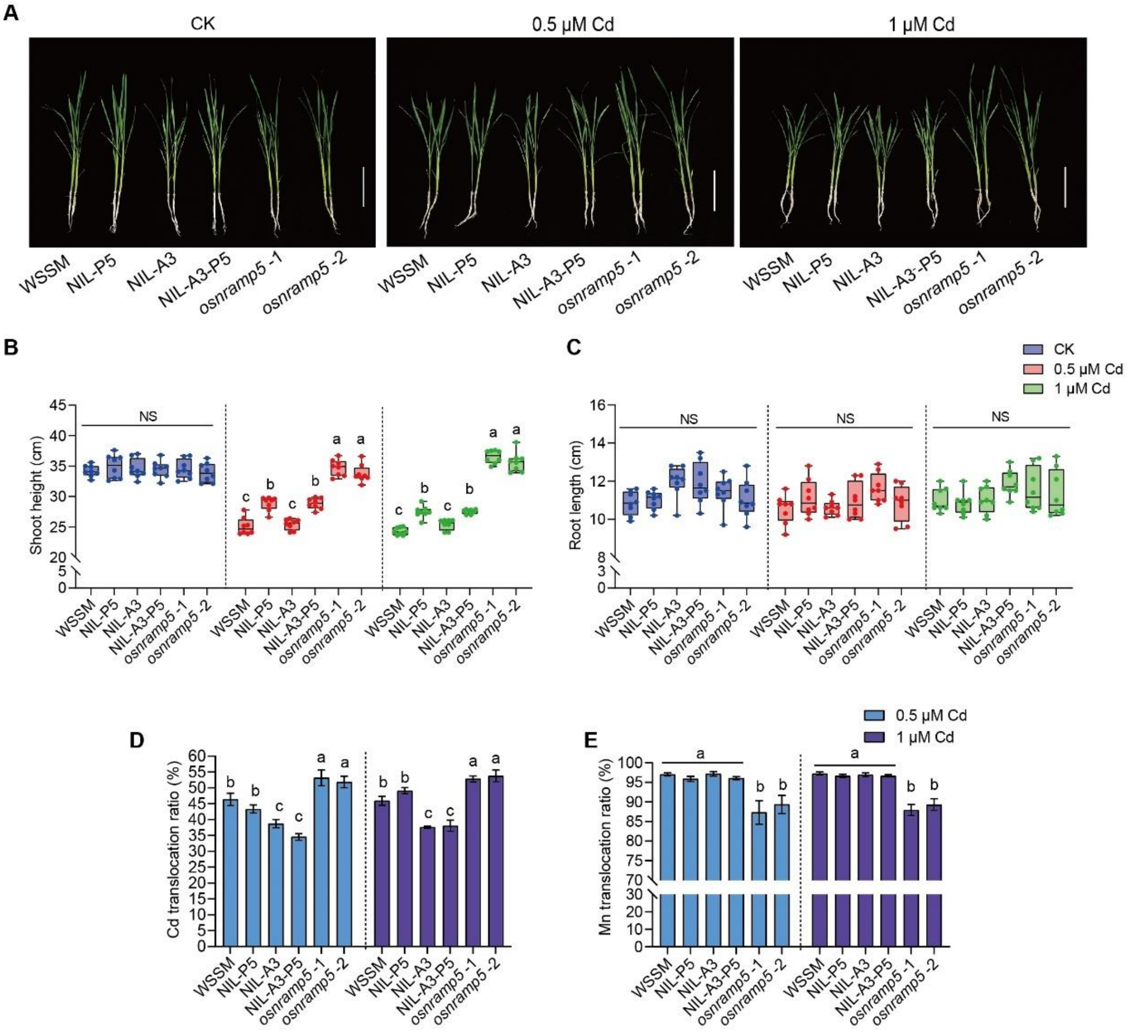

Supplemental Figure 5. Comparison of growth and metal translocation ratio among NILs, *osnramp5*, and the wild type plants in response to Cd exposure.
(A–C) The wild type (WSSM), *osnramp5* mutant, NIL-*OsNRAMP5*^{LAA} (NIL-P5), NIL-*OsHMA3*^{LAA} (NIL-A3), and NIL-*OsHMA3*^{LAA}-*OsNRAMP5*^{LAA} (NIL-A3-P5) seedlings were treated as described in the legend of Figure 3 A–D. These plants were photographed **(A)**, and their shoot height **(B)** and root length **(C)** were measured after Cd treatments for 14 days. CK, untreated control with 9 μM Mn supply. Scale bar, 10 cm. Box-and-whisker plots display the minima and maxima, the 25th and 75th percentiles (box), and medians (center line) of eight biological replicates in (**B and C**).
(D and E) Ratios of Cd **(D)** and Mn **(E)** translocation from roots to shoots. Values are presented as means ± SD of three biological replicates in **(D and E)**.
Different lowercase letters indicate significant differences by one-way ANOVA with Tukey's test ($P < 0.05$). NS, not significant.

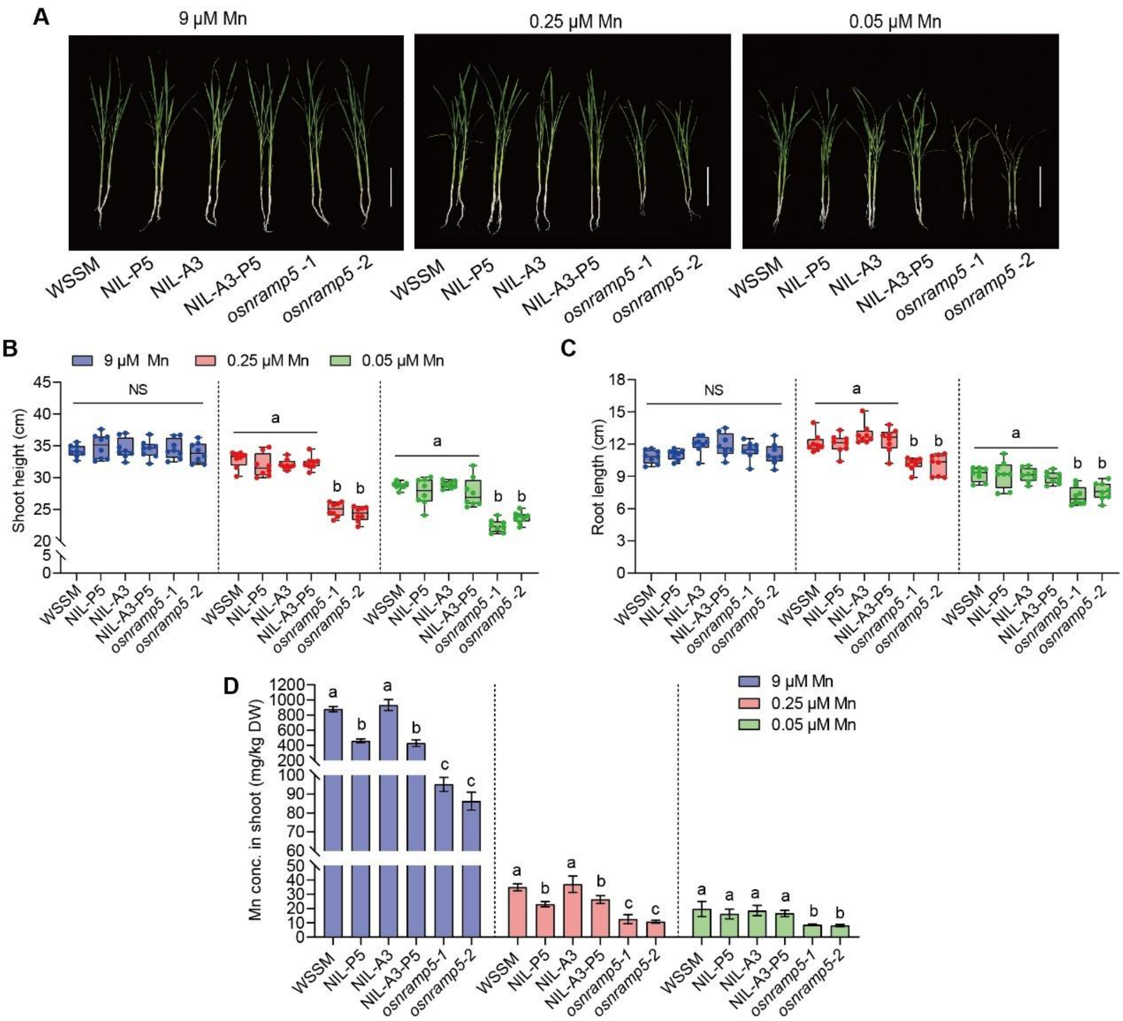

Supplemental Figure 6. Comparison of growth and shoot Mn concentrations in NILs, *osnramp5* and the wild type plants in response to different Mn supply.

(A–C) Seeds of the wild type (WSSM), *osnramp5* mutant, NIL-*OsNRAMP5*^{LAA} (NIL-P5), NIL-*OsHMA3*^{LAA} (NIL-A3), and NIL-*OsHMA3*^{LAA} -*OsNRAMP5*^{LAA} (NIL-A3-P5) were grown in a nutrient solution containing 9 or 0.25 μM Mn for 28 days or in standard nutrient solution (9 μM Mn) for 3 days and then transferred to a nutrient solution with 0.05 μM Mn for 25 days. These plants were photographed **(A)**, and their shoot height **(B)** and root length **(C)** were measured. Scale bar, 10 cm. Box-and-whisker plots display the minima and maxima, the 25th and 75th percentiles (box), and medians (center line) of eight biological replicates in (**B and C**).

(D) Shoot Mn concentrations of plants in the presence of 9, 0.25, and 0.05 μM Mn. Values are presented as means ± SD of three biological replicates.

Different lowercase letters indicate significant differences by one-way ANOVA with Tukey's test (P < 0.05). NS, not significant.

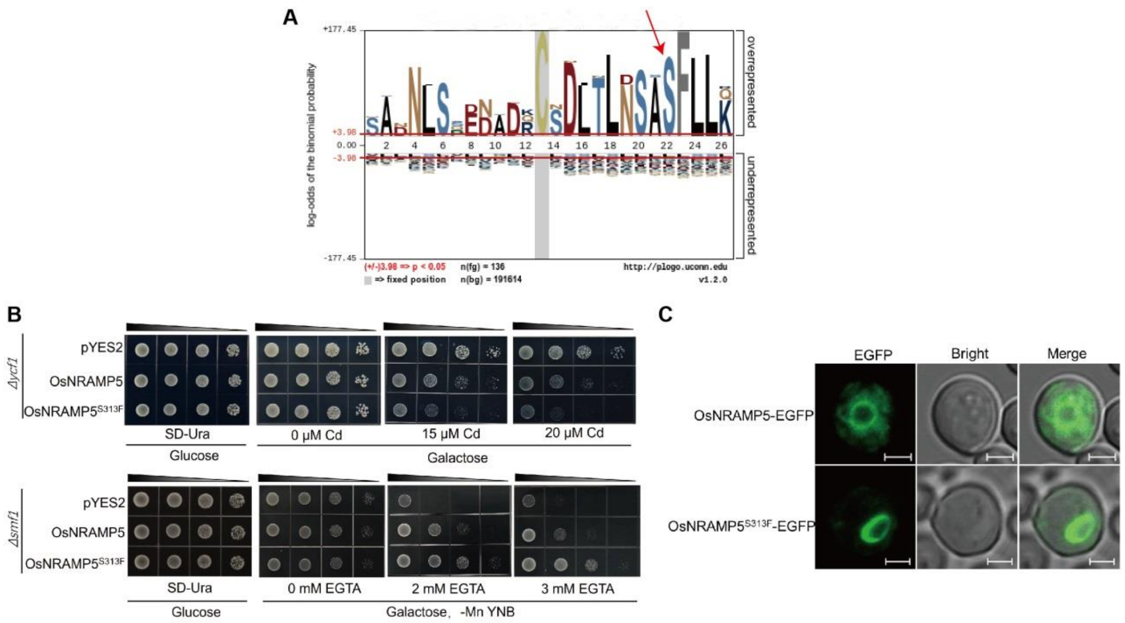

Supplemental Figure 7. Effects of *OsNRAMP5* and *OsNRAMP5^{S313F}* expression on yeast growth.

(A) Sequence conservation analysis of 250 loop homologs from different species shows that the S313 amino acid residues are conserved. The S313 residue is indicated by a red arrow.

(B) Growth phenotypes of yeast strains expressing OsNRAMP5 and OsNRAMP5^{S313F}. This experiment was conducted according to the method described in the legend of Figure 4 C.

(C) Subcellular localization of OsNRAMP5-EGFP and OsNRAMP5^{S313F}-EGFP in yeast cells. Scale bar, 2 µm.

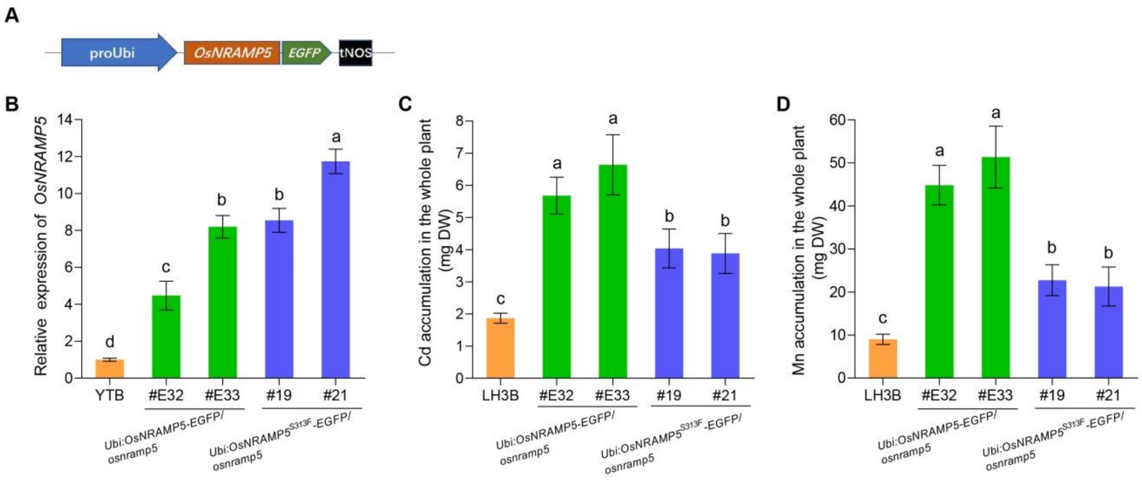

Supplemental Figure 8. Characterization of plants overexpressing *OsNRAMP5-EGFP* or *OsNRAMP5^{S313F}-EGFP* in *osnramp5* background.

(A) Schematic representation of *OsNRAMP5-EGFP* overexpression vectors.

(B) Determination of gene expression level in roots of plants overexpressing *OsNRAMP5-EGFP* or *OsNRAMP5^{S313F}-EGFP*. The transcript levels were determined by quantitative RT-PCR and calculated relative to those in the YTB (wild-type plants).

(C and D) Comparison of Cd **(C)** and Mn **(D)** accumulation among plants overexpressing *OsNRAMP5-EGFP* and *OsNRAMP5^{S313F}-EGFP*, and LH3B (*osnramp5*).

Values are presented as means ± SD of three biological replicates in **(B–D)**. Different lowercase letters indicate significant differences by one-way ANOVA with Tukey's test ($P < 0.05$).

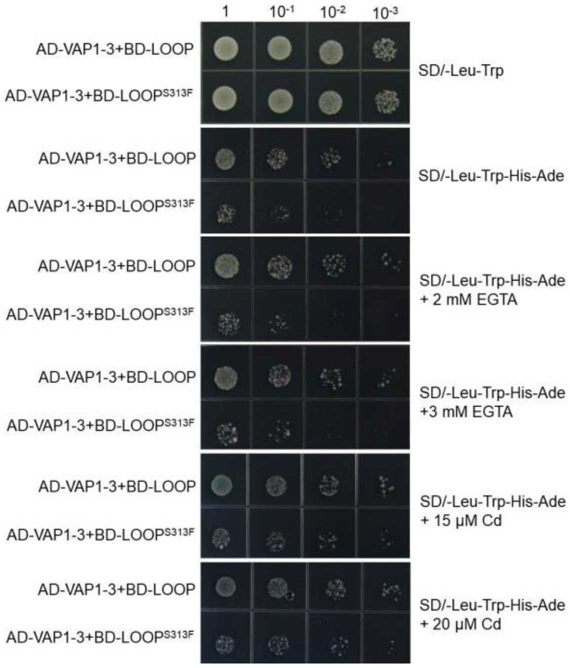

Supplemental Figure 9. Y2H assay of interaction between Loop or Loop^{S313F} and OsVAP1-3 under low-Mn conditions or different Cd supplementations.

Yeast transformants were grown on SD (-Leu/Trp) and SD (-Leu/Trp/His/Ade) media supplemented with 2, 3 mM EGTA (Mn^{2+} chelator) or 15, 20 μM Cd.

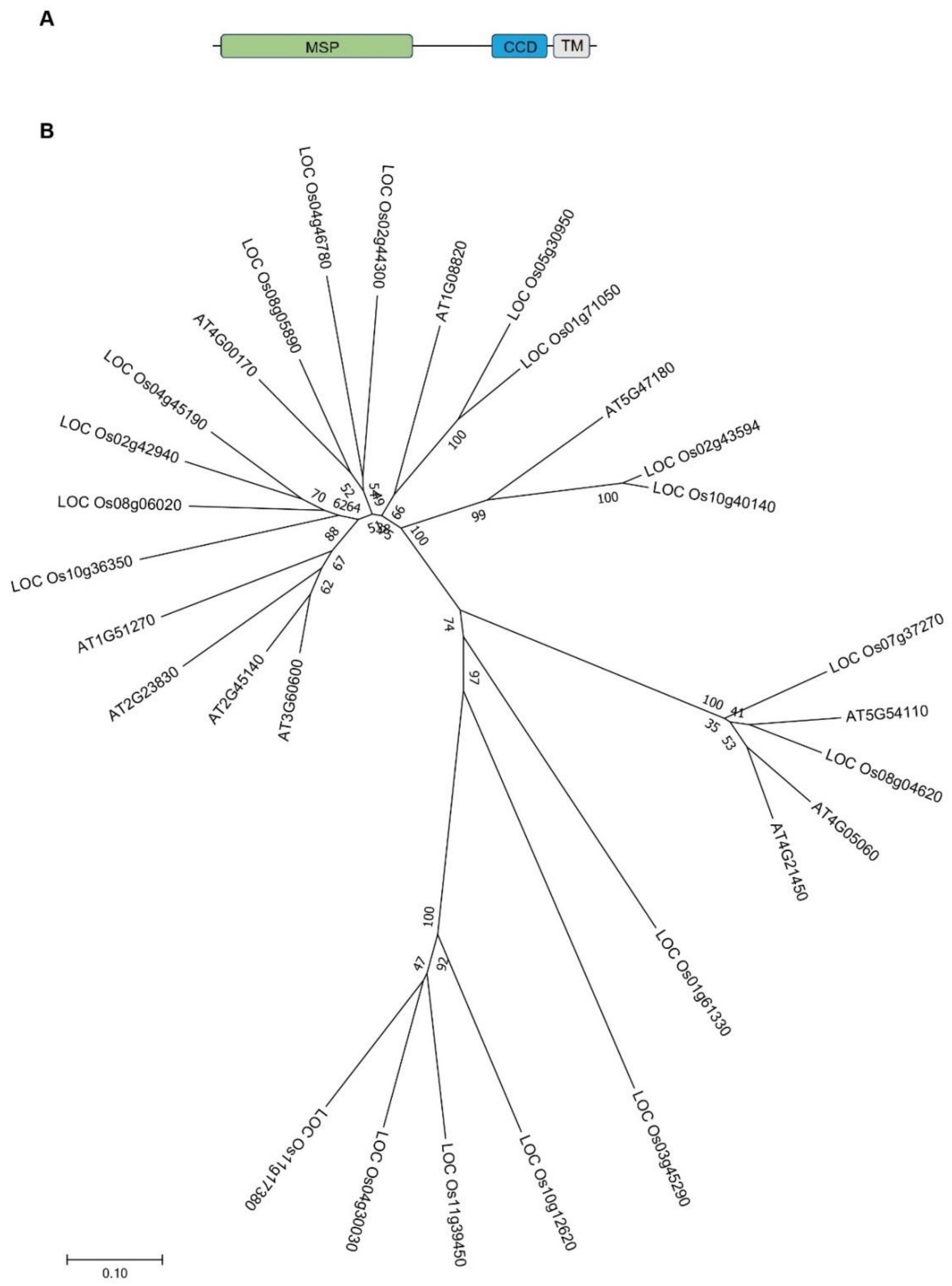

Supplemental Figure 10. Domain composition of OsVAP1-3 and phylogenetic analysis of VAPs in rice and *Arabidopsis*.

(A) Schematic representation of predicted domains in OsVAP1-3 protein. MSP, major sperm domain; CCD, coiled-coil domain; TMD, transmembrane domain.

(B) Phylogenetic analysis of VAPs proteins from rice (Os) and VAPs proteins from *Arabidopsis* (AT). The phylogenetic tree was constructed with MEGA software (Version 7.0) using the neighbor-joining (NJ) method (1000 replicates).

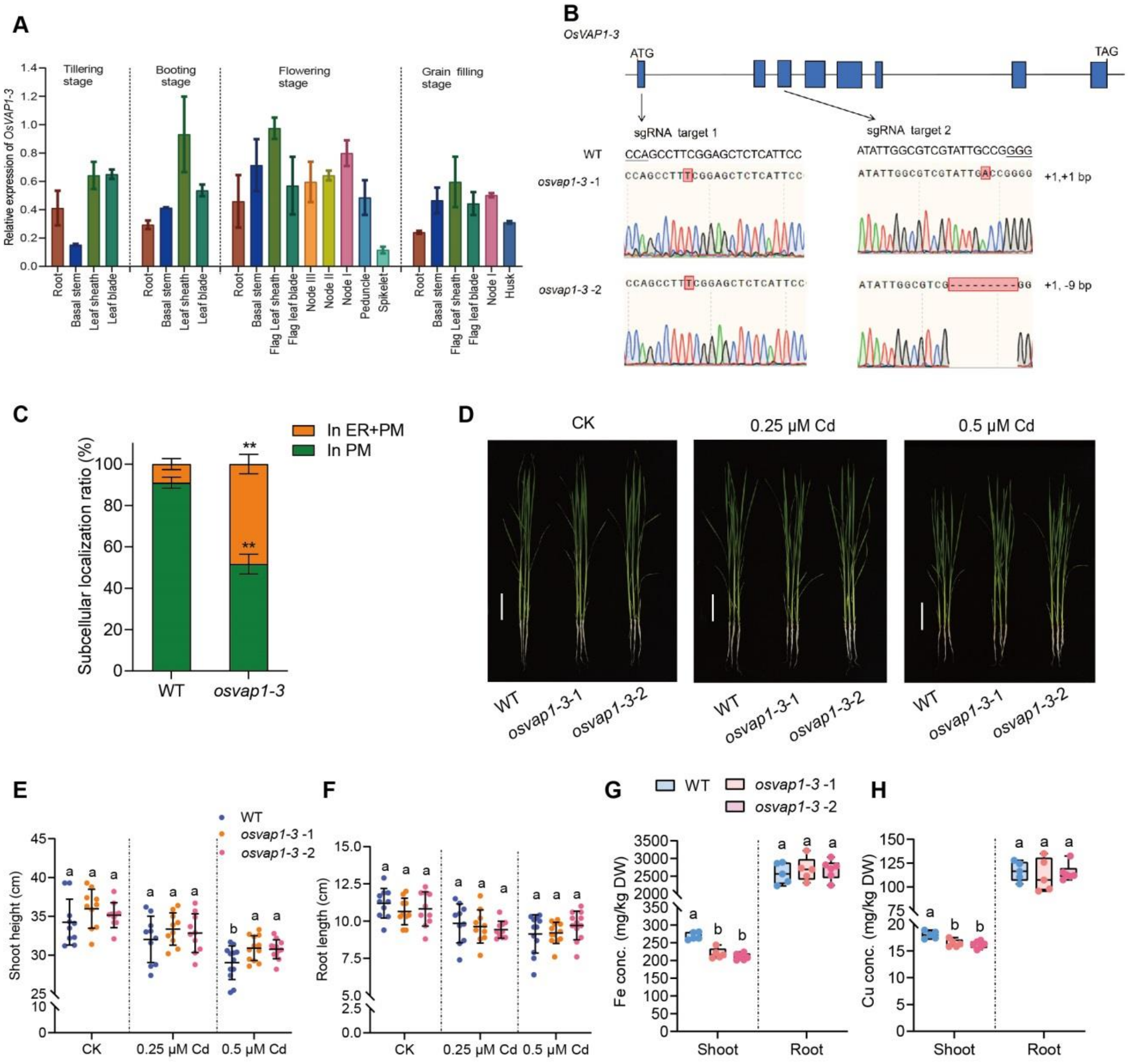

Supplemental Figure 11. Expression pattern of *OsVAP1-3* and characteristics of *osvap1-3* mutants.

(A) Relative expression levels of *OsVAP1-3* in various tissues at different growth stages. Tissues were sampled from HZ, grown in a paddy field. The transcript levels were determined by quantitative RT-PCR and calculated relative to *OsActin* 1 (Os03g0718100) expression levels. Values are presented as means ± SD of three biological replicates.

(B) Schematic representation of *OsVAP1-3* gene structure and identification of *osvap1-3* mutants in WSSM background.

(C) Quantification of subcellular localization of OsNRAMP5 in the WT and *osvap1-3* protoplasts. Values are presented as means ± SD of three biological replicates. More than 100 protoplasts were observed in each biological replicate. Two asterisks indicate that the percentage of *osvap1-3* protoplasts and WT protoplasts with the same localization differed significantly by Student's *t*-test ($P < 0.01$).

(D–H) The *osvap1-3* mutants and WT seedlings were grown in the standard nutrient solution for 2 weeks and then exposed to 0, 0.25, and 0.5 µM Cd for additional 2 weeks. These plants were photographed **(D)**, and their shoot height **(E)**, root length

(F), and metal concentrations **(G and H)** were determined. CK, untreated control. Scale bar, 10 cm. Fe **(G)** and Cu **(H)** concentrations in shoots and roots of *osvap1-3* mutants and WT plants exposed to 0.5 μM Cd are shown. Values are presented as means ± SD of ten to twelve biological replicates in **(E and F)**. Box-and-whisker plots display the minima and maxima, the 25th and 75th percentiles (box), and medians (center line) of five to six biological replicates in **(G and H).** Different lowercase letters indicate significant differences by one-way ANOVA with Tukey's test (P < 0.05).

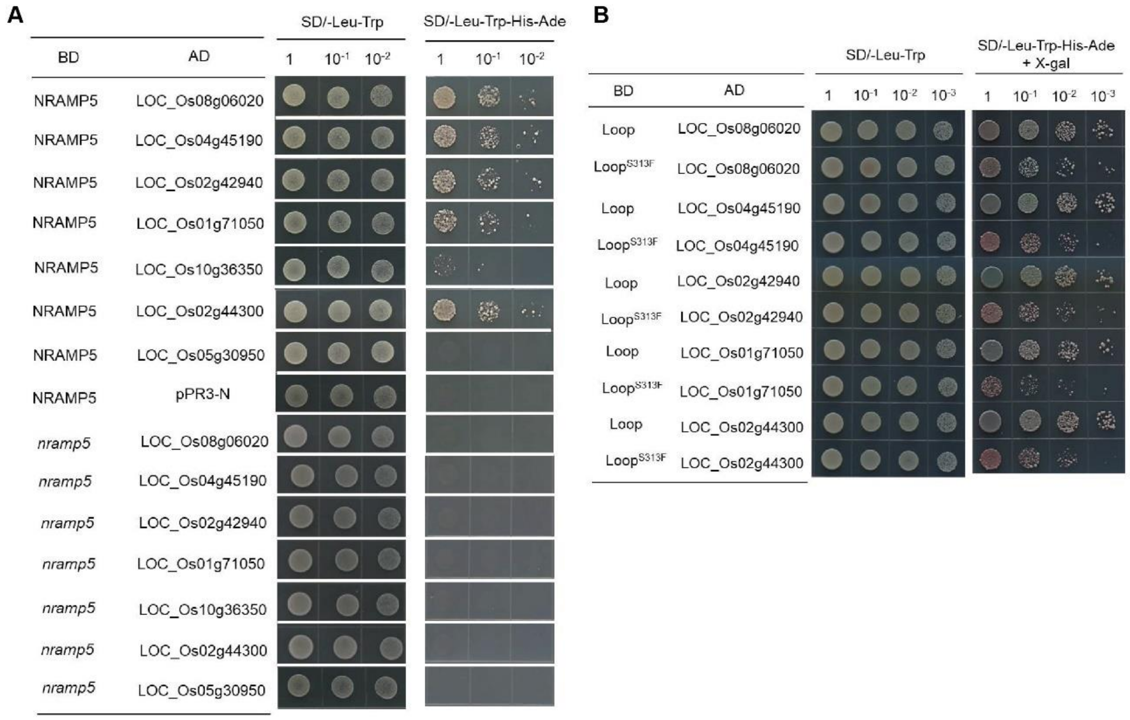

Supplemental Figure 12. Assay of interactions between NRAMP5/Loop/Loop^{S313F} and VAPs from rice via split-ubiquitin membrane-based Y2H system.

(A) Y2H assay of interactions between NRAMP5 and VAPs from rice.

(B) Y2H assay of interactions between Loop or Loop^{S313F} of OsNRAMP5 and VAPs from rice.

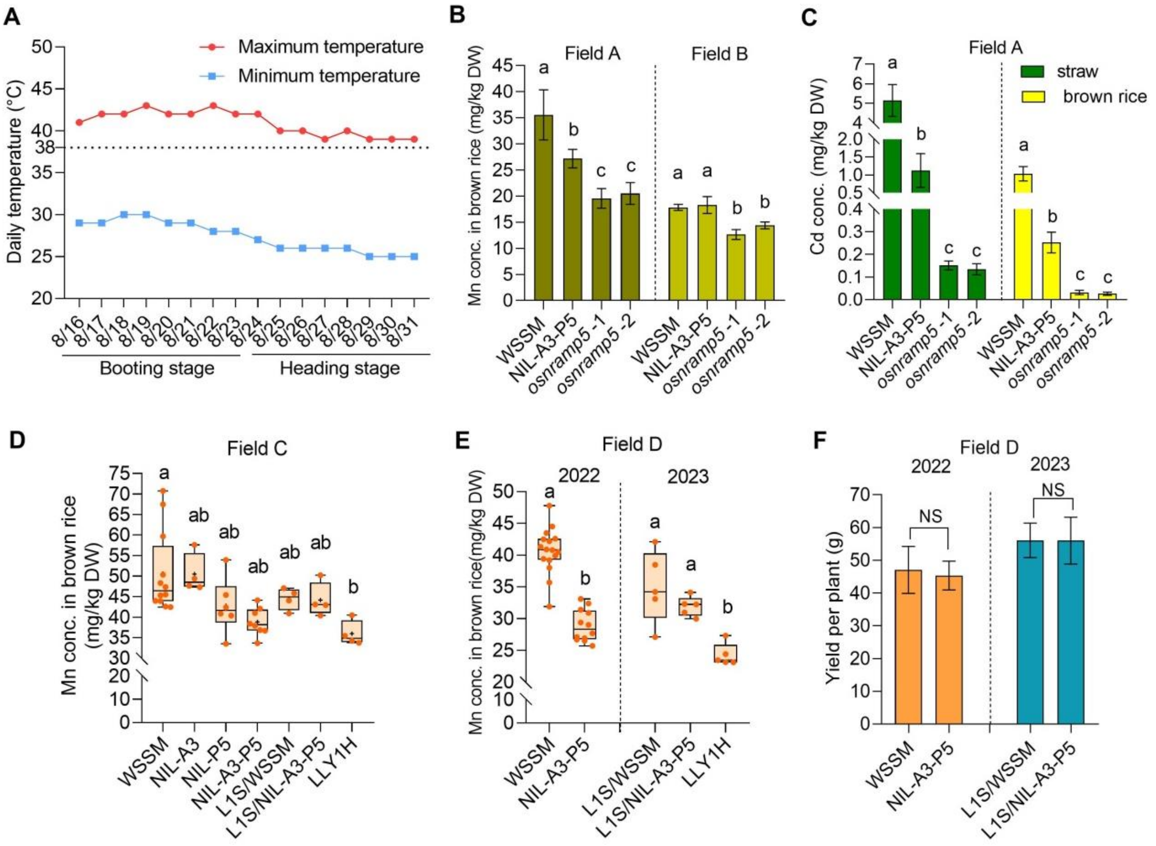

Supplemental Figure 13. Comparison of Mn concentrations among NILs, *osnramp5* and wild type plants under paddy field conditions.

(A–C) Plants of NIL-*OsHMA3*^{LAA}-*OsNRAMP5*^{LAA} (NIL-A3-P5), *osnramp5* and the wild type (WSSM) were cultivated in fields A and B as described in the legend of Figure 6 A–I. The temperatures in the field during the booting and heading stages of rice were recorded **(A)**. The Mn concentrations in brown rice **(B)** and Cd concentrations in straw and brown rice **(C)** were determined at maturity.

(D–F) NILs and their hybrid rice were cultivated in fields C and D as described in the legend of Figure 6 J–P. The brown rice Mn concentrations in Field C **(D)** and Field D **(E)**, and yield per plant in Field D **(F)** were determined at maturity.

Values are presented as means ± SD of four biological replicates in **(B, C, F)**. Box-and-whisker plots display the minima and maxima, the 25th and 75th percentiles (box), and medians (center line) of four to sixteen biological replicates in **(D and E)**. Different lowercase letters indicate significant differences by one-way ANOVA with Tukey's test ($P < 0.05$). NS indicates no significant difference by Student’s *t*-test ($P <$ 0.05).

Supplemental tables

Supplemental Table 1. Information regarding the 20 cultivars used in this study

Field ID	Name (Designation)	Origin	Subpopulation
Nip	Nipponbare	Japan	*Temperate japonica*
osnramp5	*osnramp5* mutant in Huazhan background	China	*Indica*
LAA	LAYANDABU	Indonesia	*Tropical japonica*
LLQ	Laolaiqing	China	*Temperate japonica*
ZH11	Zhonghua11	China	*Temperate japonica*
TJ1H	Tuanjie1hao	China	*Indica*
GH998	Guanghui998	China	*Indica*
JFZ	Jiafuzhan	China	*Indica*
MH3724	Mianhui3724	China	*Indica*
XWX12H	Xiangwanxian12hao	China	*Indica*
WYJ27	Wuyunjing27hao	China	*Temperate japonica*
CGN	Cungunuo	China	*Temperate japonica*
C105	ARC 12920	India	*Aus*
MH86	Minghui86	China	*Indica*
HHZ	Huanghuazhan	China	*Indica*
MXZ2H	Meixiangzhan2hao	China	*Indica*
WSSM	Wushansimiao	China	*Indica*
HZ	Huazhan	China	*Indica*
C46	PDR 34-2-1-2	Pakistan	*Admix*
C908	TJERE ENER	Indonesia	*Indica*

Supplemental Table 2. Primer sequences of KASP markers

Marker name	Primer name	Primer sequence (5'-3')
M297	M297-F1	GAAGGTGACCAAGTTCATGCTTGTTTTGCAGATTTTCATGGTC
	M297-F2	GAAGGTCGGAGTCAACGGATTTGTTTTGCAGATTTTCATGGTG
	M297-R	CAAATGAGTGGGGTTAATCATTCA
M298	M298-F1	GAAGGTGACCAAGTTCATGCTCCTAAGACCGCGAGAGGCC
	M298-F2	GAAGGTCGGAGTCAACGGATTCCTAAGACCGCGAGAGGCT
	M298-R	ACAAGCCACGACAGCCTTCAT
M299	M299-F1	GAAGGTGACCAAGTTCATGCTGTTGCTTATCTGTTGTCTTTCGAAGG
	M299-F2	GAAGGTCGGAGTCAACGGATTGTTGCTTATCTGTTGTCTTTCGAAGA
	M299-R	CATGACCCAATATTTCCTTGGGAGTT
M300	M300-F1	GAAGGTGACCAAGTTCATGCTTAAACCCTAGCTCTCTTAGCCGT
	M300-F2	GAAGGTCGGAGTCAACGGATTTAAACCCTAGCTCTCTTAGCCGA
	M300-R	GCCATCCAGAATATAATAGACTCTACTAGAG
M302	M302-F	CGAACACTTGGACTACTCTACCATTT
	M302-R1	GAAGGTGACCAAGTTCATGCTTGGGAATAGGAGGAAAACTAAACACG
	M302-R2	GAAGGTCGGAGTCAACGGATTTGGGAATAGGAGGAAAACTAAACACA
M303	M303-F1	GAAGGTGACCAAGTTCATGCTAGATACTCGTGCTGCCTGCTAA
	M303-F2	GAAGGTCGGAGTCAACGGATTAGATACTCGTGCTGCCTGCTAG
	M303-R	GCAGTTCCAATGACAATGAGTAATAGTAGT
M938	M938-F	GACAAGTGCGCCAACCTCAG
	M938-R1	GAAGGTGACCAAGTTCATGCTGAATGAATGACCTTGAGAAGGAAGA
	M938-R2	GAAGGTCGGAGTCAACGGATTGAATGAATGACCTTGAGAAGGAAGG

Supplemental Table 3. Sequences of primers used in this study

Note	Primer name	Primer sequence (5'-3')
Real-time PCR	*OsActin1*-qPCR-F	CAACACCCCTGCTATGTACG
	OsActin1-qPCR-R	CATCACCAGAGTCCAACACAA
	OsNRAMP5-qPCR-F	AGCAGCAGTAAGAGCAAGATGG
	OsNRAMP5-qPCR-R	GGGGAGGTCGTTGTGGATG
	OsHMA3-qPCR-F	GGAGGTGTCCGATTCTTCG
	OsHMA3-qPCR-R	TTTCACTTCACCGGAGTTCATC
	OsVAP1-3-qPCR-F	GAGAGGCCCACAGAGCCATC
	OsVAP1-3-qPCR-R	GGGAGAAACCGCCACTGCTT
Subcellular localization	pYBA1132-*OsNRAMP5*-F	cgcggtggcggccgctctagaATGGAGATTGAGAGAGAGAGCAGTG
	pYBA1132-*OsNRAMP5*-R	gataagcttgatatcgaattcCCTTGGGAGCGGGATGTC
	pYBA1132-*OsVAP1-3*-F	cgcggtggcggccgctctagaATGAGCGCGAGTTTCCTCG
	pYBA1132-*OsVAP1-3*-R	gataagcttgatatcgaattcTGTCTTCTTGAGGATGTAGCCCA
	p*Ubi::OsNRAMP5-eGFP*-F	gtgttacttgagctcggtaccATGGAGATTGAGAGAGAGAGCAGTG
	p*Ubi::OsNRAMP5-eGFP*-R	aatgtttgaacgatcctgcagGCGGCCGCTTTAAGATCTG
Assay in yeast	pYES2-*OsNRAMP5*-F	gggaatattaagcttggtaccATGGAGATTGAGAGAGAGAGCAGTG
	pYES2-*OsNRAMP5*-R	tgatggatatctgcagaattcCTACCTTGGGAGCGGGATG
	pYES2-*OsNRAMP5-eGFP*-F	gggaatattaagcttggtaccATGGAGATTGAGAGAGAGAGCAGTG
	pYES2-*OsNRAMP5-eGFP*-R	tgatggatatctgcagaattcTTACTTGTACAGCTCGTCCATGCC
	pYES2-*OsIST2C*-F	tcacactggcggccgctcgagAAATCTTCCCATGATGACGTTGC
	pYES2-*OsIST2C*-R	tacatgatgcggccctctagaTTAAAGCTTCTTTTTCAGCTTATGCA
	pYES2-*OsNRAMP5-OsIST2C*-F	gggaatattaagcttggtaccATGGAGATTGAGAGAGAGAGCAGTG
	pYES2-*OsNRAMP5-OsIST2C*-R	tgatggatatctgcagaattcCCTTGGGAGCGGGATGTC
	pYES2-*OsNRAMP5-eGFP-OsIST2C*-F	gggaatattaagcttggtaccATGGAGATTGAGAGAGAGAGCAGTG
	pYES2-*OsNRAMP5-eGFP-OsIST2C*-R	tgatggatatctgcagaattcCTTGTACAGCTCGTCCATGCC

Note	Primer name	Primer sequence (5'-3')
	pBT-N-Loop-F	atcgaattcctgcagggccattacggccATGGCATCAGTCAGAGGAATCAA
	pBT-N-Loop-R	agctacttaccatggggccgaggcggccCTACTTGAGAAGGAAGGAGGAGGTG
	pPR3-N-*OsVAP1-3*-F	gtatcaacgcagagtggccattacggccATGAGCGCGAGTTTCCTCG
	pPR3-N-*OsVAP1-3*-R	atcgaattctcgagaggccgaggcggccCTATGTCTTCTTGAGGATGTAGCCC
	pBT-N-*OsNRAMP5*-F	atcgaattcctgcagggccattacggccATGGAGATTGAGAGAGAGAGCAGTG
	pBT-N-*OsNRAMP5*-R	agctacttaccatggggccgaggcggccCTACCTTGGGAGCGGGATG
	pPR3-N-LOC_Os08g06020-F	gtatcaacgcagagtggccattacggccATGGCTTCCCCCGGACCG
	pPR3-N-LOC_Os08g06020-R	atcgaattctcgagaggccgaggcggccTTATGACTTCATCATATAGCCCAAGAA
	pPR3-N-LOC_Os04g45190-F	gtatcaacgcagagtggccattacggccATGGGCTCCGACTCCAAGG
Y2H	pPR3-N-LOC_Os04g45190-R	atcgaattctcgagaggccgaggcggccCTATCTCTTCATGATATACCCCAGTAGAA
	pPR3-N-LOC_Os02g42940-F	gtatcaacgcagagtggccattacggccATGCAGGAGGCGAAAAAAGA
	pPR3-N-LOC_Os02g42940-R	atcgaattctcgagaggccgaggcggccTCATCTCTTAATGAGAAAACCAAGCA
	pPR3-N-LOC_Os01g71050-F	gtatcaacgcagagtggccattacggccATGGGCCAGGACCTCGCC
	pPR3-N-LOC_Os01g71050-R	atcgaattctcgagaggccgaggcggccTCATAAGCGCAGGAGGTAACCC
	pPR3-N-LOC_Os10g36350-F	gtatcaacgcagagtggccattacggccATGGCCGCCTCCTGCGAC
	pPR3-N-LOC_Os10g36350-R	atcgaattctcgagaggccgaggcggccTCATTTCTTTACAAGACAACCCAGA
	pPR3-N-LOC_Os02g44300-F	gtatcaacgcagagtggccattacggccATGAGTAACACCCTGCTTCGAATC
	pPR3-N-LOC_Os02g44300-R	atcgaattctcgagaggccgaggcggccCTAAACATTGCTTCCCGCCA
	pPR3-N-LOC_Os05g30950-F	gtatcaacgcagagtggccattacggccATGGGCAGCGTGGACTTCG
	pPR3-N-LOC_Os05g30950-R	atcgaattctcgagaggccgaggcggccTCATAGGTGCAACAGGTAACCG

Note	Primer name	Primer sequence (5'-3')
BiFC	p*OsNRAMP5*-cYFP-F	tctaggagctcggtacccgggATGGAGATTGAGAGAGAGAGCAGTG
	p*OsNRAMP5*-cYFP-R	atcgtatgggtacatactagtCCTTGGGAGCGGGATGTC
	pnYFP-*OsVAP1-3*-F	tctgaggaggatcttcccgggATGAGCGCGAGTTTCCTCG
	pnYFP-*OsVAP1-3*-R	agggcatgcctgcaggtcgacCTATGTCTTCTTGAGGATGTAGCCC
LUC	pcLUC-*OsVAP1-3*-F	tacgcgtcccggggcggtaccATGAGCGCGAGTTTCCTCG
	pcLUC-*OsVAP1-3*-R	acgaaagctctgcaggtcgacCTATGTCTTCTTGAGGATGTAGCCC
	pLoop-nLUC-F	acgggggacgagctcggtaccATGGCATCAGTCAGAGGAATCAA
	pLoop-nLUC-R	cgcgtacgagatctggtcgacCTTGAGAAGGAAGGAGGAGGTGT
	pLoop(S313F)-nLUC-R	cgcgtacgagatctggtcgacCTTGAGAAGGAAGAAGGAGGTGT
CoIP	p580-*OsVAP1-3*-FLAG-F	agtccggagctagctctagaATGAGCGCGAGTTTCCTCG
	p580-*OsVAP1-3*-FLAG-R	tgatctttgtaatcggatccTGTCTTCTTGAGGATGTAG
Prime editing	pegRNA-S313F	aatgaccttgagaaggaagggtttcagagctatgctggaaacagcatagcaagttgaaataaggctagtccgttatcaacttgaaaaagtggcaccgagtcggtgctcgacacctcctttttccttctcaagtaccctaattgacgcggttctatctagttacgcgttaaaccaactagaaa
	pegRNA-S313A	gaccttgagaaggaaggagggtttcagagctatgctggaaacagcatagcaagttgaaataaggctagtccgttatcaacttgaaaaagtggcaccgagtcggtgctcgacacctccgccttccttctctctcctttttgacgcggttctatctagttacgcgttaaaccaactagaaa
CRISPR/Cas9	*OsNRAMP5*-U6a-F	gccgGTTCTTCCTGTACGAGAGC
	OsNRAMP5-U6a-R	aaacGCTCTCGTACAGGAAGAAC
	OsNRAMP5-U3-F	ggcaCACCTCCTCCTTCCTTCTCA
	OsNRAMP5-U3-R	aaacTGAGAAGGAAGGAGGAGGTG
	OsVAP1-3-U6a-F	gccgGAATGAGAGCTCCGAAGGC
	OsVAP1-3-U6a-R	aaacGCCTTCGGAGCTCTCATTC
	OsVAP1-3-U3-F	ggcaTATTGGCGTCGTATTGCCG
	OsVAP1-3-U3-R	aaacCGGCAATACGACGCCAATA

Note	Primer name	Primer sequence (5'-3')
Target site sequencing	*OsNRAMP5*-F	CGGCATCAGTCAGAGGAATC
	OsNRAMP5-R	AGGACGGAGAAATCGTGTAGAC
	OsVAP1-3-F	GATCGAATCCGGTGGAAAGC
	OsVAP1-3-R	AGGTGCCTCCCTCTGTGCTT
